# Balancing inflammatory signaling in hematopoiesis: roles of MIRC11 and miR-146a

**DOI:** 10.3389/fimmu.2026.1828453

**Published:** 2026-07-08

**Authors:** Austin C. Boucher, Thomas J. Payne, Kirthi Pulakanti, Sridhar Rao, Richard Dahl

**Affiliations:** 1 Department of Biological Sciences, University of Notre Dame, Notre Dame, IN, United States; 2 Harper Cancer Research Institute, Notre Dame, IN, United States; 3 Department of Microbiology and Immunology, Indiana University School of Medicine, South Bend, IN, United States; 4 Blood Research Institute, Versiti, Milwaukee, WI, United States; 5 Department of Cell Biology, Medical College of Wisconsin, Milwaukee, WI, United States; 6 Department of Pediatrics, Medical College of Wisconsin, Milwaukee, WI, United States

**Keywords:** hematopoietic stem cell, inflammation, macrophage, microRNA, mir-146a, MIRC11

## Abstract

**Introduction:**

Inflammatory signaling regulates hematopoietic stem and progenitor cell (HSPC) function, but mechanisms that maintain inflammatory signaling within a physiological range remain incompletely understood. MicroRNAs (miRNAs) are important regulators of immune signaling pathways. In this report we hypothesized that MIRC11 miRNAs (miR-23a, miR-24-2, and miR-27a) and miR-146a which oppositely regulate NF-κB activity, function to fine-tune inflammatory responses in hematopoietic cells.

**Methods:**

Inflammatory gene expression in *mmu-Mirc11^-/-^* HSPCs was analyzed by RNA sequencing. To examine functional interactions between mmu-MIRC11 and mmu-miR-146a miRNAs *in vivo*, we generated *Mirc11^-/-^Mir146a^-/-^* double knockout mice. Hematopoietic populations were characterized by flow cytometry, and inflammatory responses were assessed in bone marrow–derived macrophages following Toll-like receptor (TLR) stimulation.

**Results:**

RNA sequencing revealed reduced activation of inflammatory pathways including interferon, TNF, IL-6, and TLR signaling in mmu-MIRC11-deficient HSPCs. Double knockout mice were viable and exhibited modest changes in the lineage skewing observed in *Mirc11^-/-^* mice but did not rescue the progressive bone marrow failure associated with mmu-miR-146a deficiency. In macrophages, combined loss of mmu-MIRC11 and mmu-miR-146a produced gene-specific effects on inflammatory responses, frequently enhancing cytokine expression compared with single mutants.

**Discussion:**

These findings demonstrate that MIRC11 promotes inflammatory signaling in hematopoietic cells and that interactions between MIRC11 and miR-146a regulate inflammatory gene expression in a context-dependent manner. Together, these results highlight the complexity of miRNA-mediated control of immune signaling and suggest that coordinated manipulation of miRNA pathways may enable selective modulation of inflammatory responses.

## 1 Introduction

Hematopoietic stem cells (HSCs) sustain lifelong blood production through tightly regulated processes of quiescence, self-renewal, and differentiation (1). These processes are highly responsive to inflammatory signals generated during infection, tissue damage, and immune activation (2, 3). Cytokines and pattern-recognition receptor pathways—including Toll-like receptor (TLR), interferon (IFN), tumor necrosis factor (TNF), and interleukin-1 (IL-1) signaling—can directly influence HSC activity and lineage specific differentiation (4–9). During acute inflammatory responses, HSCs shift from a predominantly quiescent state toward proliferation and differentiation, particularly toward myeloid lineages that support innate immune defense. However, prolonged or dysregulated inflammatory signaling can disrupt hematopoietic homeostasis and contribute to pathological conditions including bone marrow failure (BMF), myelodysplastic syndrome (MDS), and acute myeloid leukemia (AML) (10).

Despite the pathogenic effects of excessive inflammation, basal inflammatory signaling is also required for normal hematopoiesis (11). Low-level activation of inflammatory pathways contributes to steady-state HSC proliferation and lineage output. For example, interferon signaling promotes HSC proliferation under homeostatic conditions, and microbiota-derived signals that activate TLR pathways support granulopoiesis in the bone marrow (4, 12). Genetic models affecting components of the NF-κB signaling pathway further demonstrate that moderate inflammatory signaling is necessary for maintaining HSC function and hematopoietic homeostasis (11, 13–17). Together, these findings highlight the importance of maintaining inflammatory signaling within a narrow physiological range. However, the molecular mechanisms that regulate this balance in hematopoietic stem and progenitor cells (HSPCs) remain incompletely defined.

MicroRNAs (miRNAs) are short non-coding RNAs that regulate gene expression post-transcriptionally and play important roles in controlling immune and inflammatory signaling networks (18). Several miRNAs are observed to both regulate inflammatory signaling and HSC development (19). Pro-inflammatory miRNAs (20–23) as well as anti-inflammatory miRNAs (24–27) are critical for proper HSC homeostasis. In addition to their roles in stem and progenitor cells, miRNAs also regulate the functional responses of mature immune cells such as macrophages. Macrophage activation and polarization are accompanied by dynamic changes in miRNA expression profiles, with distinct miRNAs contributing to the regulation of pro-inflammatory and anti-inflammatory gene programs (28–30).

Previously we reported that paralog miRNA cluster genes *mmu*-*Mirc11* (codes for miRNAs mmu-miR-23a, -27a, and -24-2) and *mmu*-*Mirc22* (codes for mmu-miR-23b, -27b and -24-1) are required for the maintenance of bone marrow HSCs (31). Exogenous expression of mmu-MIRC11 in murine hematopoietic progenitors promotes monocyte and granulocyte differentiation (32). In contrast, *Mirc11^-/-^* mice exhibit increased bone marrow production of B cells and their progenitors accompanied by a corresponding decrease in CD11b+ myeloid cells and their progenitors (33). The mmu-MIRC11 induced myeloid skewing phenocopies what is observed with activated inflammatory signaling in HSPCs (3). A key mediator of inflammatory myeloid skewing at the expense of lymphopoiesis is sustained activation of the transcription factor NF-κB (34). MIRC11 miRNAs target negative regulators of NF-κB (35–37).

In contrast, miR-146a functions as a well-characterized negative regulator of inflammatory signaling. MiR-146a is induced in response to inflammatory stimuli and acts as a feedback inhibitor of NF-κB signaling by targeting key signaling intermediates such as TRAF6 (38). Mice lacking mmu-miR-146a develop chronic inflammation and progressive hematopoietic abnormalities, including myeloproliferative disorders and bone marrow failure (39, 40). These phenotypes are associated with enhanced inflammatory signaling in hematopoietic progenitors and dysregulation of immune pathways.

In this study, we investigated the interaction between mmu-MIRC11 miRNAs and mmu-miR-146a in the regulation of inflammatory signaling and hematopoiesis. RNA-sequencing analysis of Mirc11-deficient murine hematopoietic stem and progenitor cells revealed reduced activation of inflammatory pathways, including interferon, TNF, and TLR signaling networks. MIRC11 miRNAs and miR-146a represent potentially opposing regulators of NF-κB and inflammatory signaling. MIRC11 promotes myeloid differentiation and can support sustained activation of NF-κB and promotes inflammatory responses, whereas miR-146a acts as a negative feedback regulator of NF-κB signaling to limit inflammation. To determine whether the products of these miRNA genes functionally interact *in vivo*, we generated *Mirc11^-/-^Mir146a^-/-^* double-knockout mice and examined their hematopoietic phenotypes. In addition, we analyzed Toll-like receptor/NF-κB–mediated inflammatory responses in bone marrow–derived macrophages from single and double mutant mice. These experiments were designed to determine whether MIRC11 and miR-146a cooperate to fine-tune inflammatory signaling in hematopoietic cells and thereby regulate hematopoietic homeostasis and immune responses.

## 2 Materials and methods

### 2.1 Animals

*Mirc11^-/-^* mice (B6.Cg-Mirc11tm1Rdhl/J) were previously generated by our laboratory (33). *Mir146a^-/-^* (B6.Cg-Mir146tm1.1Bal/J) were obtained from Jackson Laboratory (Bar Harbor, ME) (39). Both strains were on a C57BL/6J background. Double knockout mice were generated through interbreeding the mutant strains in the Freimann Life Science Center (University of Notre Dame). Mice were genotyped by Transnetyx (Memphis, TN). Mice were euthanized in an Euthanex (E-Z Systems Corporation, Palmer, PA, USA) automated induction chamber using CO_2_ gas. The chamber was filled with ~100% CO2 at a displacement rate of ~50% of the chamber volume per minute, in accordance with the AVMA Guidelines for the Euthanasia of Animals. The Euthanex was programmed for a 7-minute fill time, 5-minute dwell time and a 2-minute exhaust. After the completion of the cycle death was confirmed via cervical dislocation. Femurs and tibias were isolated as a source of bone marrow for flow cytometric analysis and for preparation of bone marrow derived macrophages. The use of mice in these experiments was approved by the Indiana University School of Medicine and University of Notre Dame IACUCs (Protocols 19-09–5543 and 22-07-7328 (RD)).

### 2.2 RNA sequencing

Hematopoietic stem and progenitor cells (Lin-Sca-1+c-Kit+) were sorted from bone marrow on a FacsAria III (BD Biosciences, Franklin Lakes, NJ). ACK lysed bone marrow was incubated with biotin labeled Lineage cocktail antibodies (B220, CD11b, CD3ε, GR1, Terr119), Streptavidin-APCcy7, Sca1-FITC, c-Kit-APC. Sca1 and c-Kit antibodies were obtained from BioLegend (San Diego, CA). For each genotype (wildtype and *Mirc11^-/-^*) 3 separate RNA preps were prepared and used to build sequencing libraries. Each RNA preparation was prepared from sorted cells acquired from bone marrow isolated from 3 to 4 mice. Cells were subsequently lysed in TRIzol reagent (Thermo Fisher Scientific, Pittsburg, PA) and RNA was isolated using Qiagen miRNeasy kit (Qiagen, Valencia, CA) following manufacturer protocol. RNA-seq libraries were prepared at the University of Notre Dame Genomics and Bioinformatics Core Facility and sequenced on an Illumina NextSeq 500 as described previously (**Supplementary Methods**) (41). Pathway analysis and upstream regulator analysis was performed using Ingenuity pathway analysis (IPA, QIAGEN Inc., https://www.qiagenbioinformatics.com/products/ingenuitypathway-analysis), Biojupies (https://biojupies.cloud/), and Broad Institute Gene Set Enrichment Analysis (GSEA) software (http://software.broadinstitute.org/gsea/index.jsp).

### 2.3 Tissue culture

For co-culture of mouse HSPCs with OP9 (ATCC, Manassas, VA) cells, nucleated cells from the femur and tibia of 8-week-old mice were lineage depleted with a MACS lineage cell separation kit according to manufacturer’s instructions (Miltenyi Biotec, Auburn, CA, USA). Lineage depleted cells were cultured on 40,000 OP9 stromal cells (plated 24h before co-culture) in Iscove’s Modified Dulbecco’s Media (IMDM) supplemented with 10% defined FBS (fetal bovine serum, SeraPrime, Ft. Collins, CO), 55mM β-mercaptoethanol (BME), 50 U/ml penicillin, 50mg/mL streptomycin, 0.1mM Glutamax, 5ng/mL Flt3L, and 1ng/mL IL-7. Recombinant mouse cytokines were obtained from BioLegend (San Diego, CA, USA). Cells were transferred onto fresh OP9 cells every 3 days. To evaluate myeloid and B cell differentiation, cells were analyzed after 15 days of culture with CD19-APC and CD11b-APC/cy7 antibodies (BioLegend, San Diego, CA, USA).

For *in vitro* macrophage experiments bone marrow derived macrophages (BMDM) were prepared from bone marrow isolated from wildtype, *Mirc11^-/-^*, *Mir146a^-/-^* and *Mirc11^-/-^Mir146a^-/-^* C57BL/6 mice. Briefly, bone marrow was flushed from femurs and tibias of individual mice with sterile phosphate buffered saline pH 7.4 (PBS). Red blood cells were lysed using ACK lysis buffer (150mM NH4Cl, 10mM KHCO3, 0.1mM EDTA in PBS). After ACK lysis, marrow was plated on 10cm tissue culture plates in Dulbecco’s Modified Eagles Media (DMEM, Sigma-Aldrich, St. Louis, MO), 10% FBS (SeraPrime, Ft. Collins, CO), 50 U/ml penicillin, and 50 ug/ml streptomycin. Unless stated otherwise, cell culture media additives were obtained from Thermo Fisher Scientific (Waltham, MA). Cells were incubated at 37°C/5% CO2 for 4h. After incubation, non-adherent cells were pelleted and resuspended in DMEM, 10%FBS, 15% L929 conditioned media, 10mM HEPES buffer, and 50 U/ml penicillin, and 50 ug/ml streptomycin and plated for 7d at 37°C/5% CO2. After 7 days, macrophages were detached using TrypLE (Thermo Fisher Scientific, Waltham, MA), counted and plated at 1x10^6^ cells/ml overnight. The following day, macrophages were stimulated with either 100 ng/ml lipopolysaccharide (LPS derived from Pseudomonas aeruginosa, Sigma-Aldrich, St. Louis, MO, L8643), 50ng/ml Polyinosinic–polycytidylic (pIpC, Sigma-Aldrich, St. Louis, MO, P9582) or 100ng/ml Pam3CSK4 (InvivoGen, San Diego, CA) for indicated times.

Hematopoietic colony assays were performed as described previously with minor modifications (42, 43). Briefly, bone marrow cells were isolated from tibias and femurs of 8-week old mice. Mature erythroid cells were removed by ammonium chloride lysis. For hematopoietic colony assays 1.0 X 10^4^ nucleated bone marrow cells were plated into methylcellulose medium containing hematopoietic cytokines (Methocult GF 3434, Stem Cell Technologies, Vancouver). Colonies were counted and scored for colony type after 7 days of incubation at 37°C in 5% CO_2_ using an Olympus inverted microscope. Scoring for colony types erythroid (Burst Forming Unit-Erythroid (BFU-E), Colony Forming Unit-Erythroid (CFU-E)) and myeloid colonies (Colony Forming Unit-Granulocyte (CFU-G), Colony Forming Unit-Monocyte (CFU-M) was done by evaluating colony morphology (44, 45).

### 2.4 Reverse transcriptase -quantitative PCR

Total RNA from bone marrow derived macrophages was isolated using TRIzol reagent (Thermo Fisher Scientific, Waltham, MA), according to manufacturer’s protocol. RNA was reversed transcribed to cDNA using the High-Capacity cDNA Reverse Transcription Kit (Thermo Fisher Scientific, Waltham, MA). qPCR was performed using gene specific fluorescent probe assays, for both miRNAs and mRNAs on a CFX96 C1000 system (Bio-Rad Laboratories, Hercules, CA, USA). Relative gene expression was calculated using the comparative threshold cycle method. MiRNA expression was normalized to Sno202 snRNA expression. mRNA expression normalized to *B2m* or *Gapdh*. miRNA cDNA primers (5x) and qPCR primers (20x) were ordered from Thermo Fisher Scientific. All other primers for qPCR were ordered from IDT (Coralville, Iowa). Unless noted otherwise all RT-qPCR analysis was performed with 3 or more experimental replicates and 3 technical replicates.

### 2.5 Flow cytometry

Bone marrow cells were collected from the femurs and tibias of mice at the indicated ages. Mature erythroid cells were removed by ammonium chloride lysis. Nucleated bone marrow cells were stained to characterize B cell (CD19/B220), myeloid (CD11b), erythroid (Terr119), MEP (Lin-, Sca1-, cKit+, CD34-, FcγRIII/II-), CMP (Lin-, Sca1-, cKit+, CD34+, FcγRIII/II-), GMP (Lin-, Sca1-, cKit+, CD34+, FcγRIII/II+), and LSK (Lin-, Sca1+, cKit+) cell populations. Unless stated otherwise, all antibodies were obtained from BioLegend (San Diego, CA, USA). Prior to staining cells were incubated with mouse TruStain FcX (BioLegend). Single cell suspensions were incubated with the following combinations of antibodies: Mature cells- TER-119-FITC, B220 (RA3-6B2)-allophycocyanin (APC), CD11b (M1-70)-allophycocyanin-Cy7 (APC/Cy7), Myeloid progenitor (MEP, CMP, GMP) CD34LSK – Sca1 (D7)- lineage cocktail (CD11b (RM 2801), B220 (RA3-6B2), CD19 (6D5), GR1 (RB6-8C5), Terr119 (Ter-119) CD3e (145-2c11))-biotin, TR-streptavidin, cKit (2B8)-APC/cy7. Stained cells were subsequently assessed using Beckman Coulter FC500 Flow Cytometer (Brea, CA, USA) or Cytek Northern Lights Spectral Flow Cytometer (Fremont, CA, USA) and data was analyzed using FlowJo software (Tree Star, Ashland, OR, USA). Dead cells were removed from analysis by the FSC/SSC gating and/or exclusion of propidium iodide (Sigma-Aldrich, St. Louis, MO, USA) or Zombie Aqua positive cells (BioLegend, San Diego, CA. USA). Basis of gates was determined with the use of fluorescence minus one (FMO) control when necessary. Results are presented as standard deviation of the mean (SD) for averages of each mouse genotype.

### 2.6 Statistical analysis

Statistical data is presented as +/- standard deviation of the mean. Differences between sample groups were determined by performing multiple comparison ANOVA analysis with PRISM software, version 10.0 (GraphPad Software, La Jolla, CA).

## 3 Results

### 3.1 RNA sequencing analysis of *Mirc11^-/-^* HSPCs

#### 3.1.1 Decreased inflammatory signaling observed in murine *Mirc11^-/-^* HSPCs

**Figure 1:**
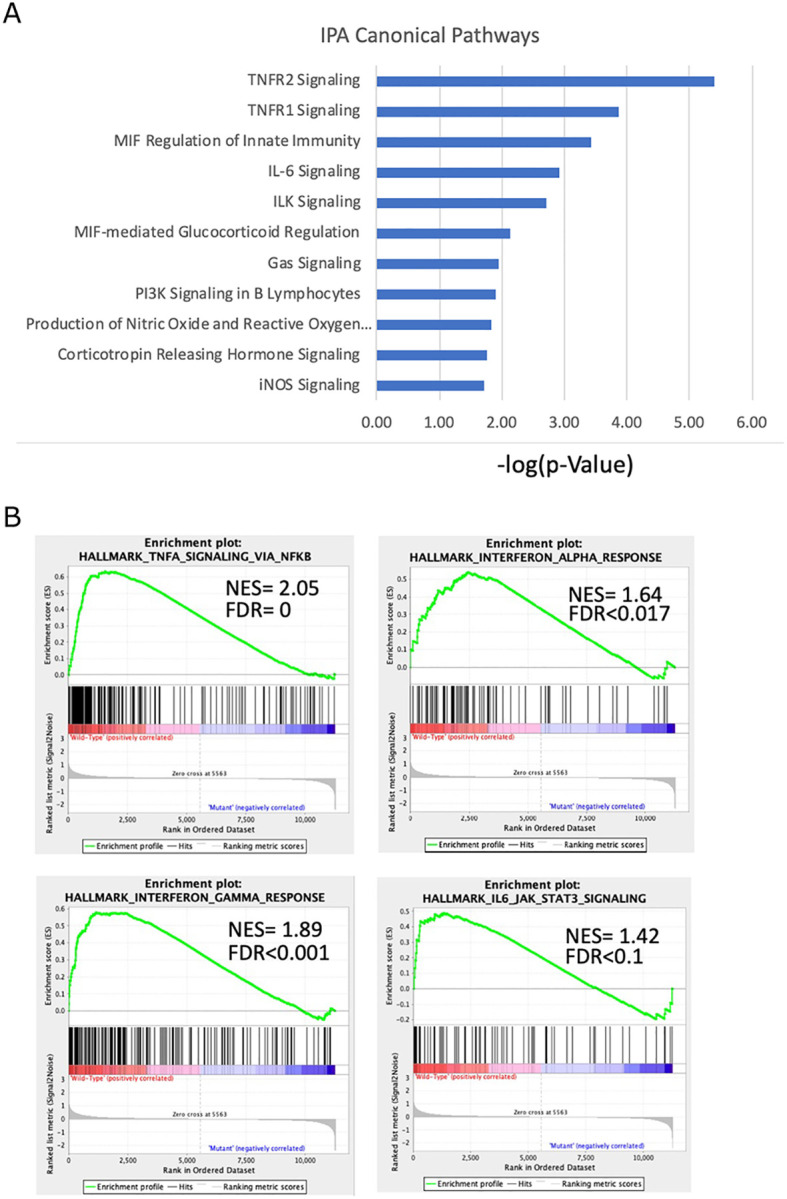
RNA-seq analysis of RNA isolated from bone marrow HSPCs reveals that *Mirc11* loss downregulates inflammatory signaling. RNA was isolated from wildtype (n=3) and *Mirc11^-/-^* (n=3) bone marrow murine HSPCs (Lin-Sca1-cKit+) and subjected to RNA-seq analysis. **(A)** Differentially expressed genes were used to identify canonical pathways affected by loss of *Mirc11* using Ingenuity Pathway Analysis (IPA, Qiagen) software. **(B)** Gene Set Enrichment Analysis (GSEA) plots of wildtype vs. *mmu*-*Mirc11^-/-^* HSPC gene expression interrogating the Molecular Signatures Database (MSigDB) Hallmark GeneSet.

We previously reported that mice lacking MIRC11 had skewed bone marrow hematopoiesis with increased B cell populations and a commensurate decrease in myeloid populations which is in part due to misregulation of PI3 kinase (PI3K)/Akt signaling and BMP/Smad signaling (33, 46). To further investigate how mmu-MIRC11 miRNAs influence hematopoietic stem and progenitor cell (HSPC) differentiation and function, we performed RNA-seq analysis comparing gene expression between wildtype and *Mirc11^-/-^* HSPCs (Lin-Sca-1+c-Kit+ bone marrow cells). Ingenuity Pathway Analysis (IPA, Qiagen) of differentially expressed genes revealed decreased TNF and IL-6 inflammatory signaling in *Mirc11^-/-^* HSPCs (**Figure 1A**). This was further supported by IPA predicted reductions in upstream regulators associated with inflammatory pathways including TNF, IL-6 and IL-1β (**Table 1**). **Supplementary Table 1** shows fold changes between wildtype and *Mirc11^-/-^* murine HSPCs for genes that are downstream of TNF, IL-6 and IL-1β signaling. In agreement with the IPA analysis, gene set enrichment analysis (GSEA) (47) identified downregulation of TNF, IFNα β γ and IL-6 signaling (**Figure 1B**). These results reinforce a common function for MIRC11 regulation of inflammatory signaling in hematopoietic cells.

**Table 1:** Upstream inflammatory pathways/molecules are downregulated in *Mirc11^-/-^* HSPCs (ingenuity pathway analysis).

Upstream regulator	Molecule type	z-score	p-value
lipopolysaccharide	chemical drug	-3.727	1.78E-18
TNF	cytokine	-2.479	1.96E-16
IL1B	cytokine	-2.805	5.05E-14
PDGF BB	complex	-3.445	1.79E-13
CREB1	transcription regulator	-3.936	6.34E-11
IL6	cytokine	-2.808	3.38E-10
forskolin	chemical toxicant	-3.193	9.03E-10
IFNG	cytokine	-4.002	4.30E-09
P38 MAPK	group	-2.897	1.20E-08
TLR9	transmembrane receptor	-2.471	4.48E-08
TLR3	transmembrane receptor	-3.164	5.73E-08
IFNAR1	transmembrane receptor	-2.18	7.49E-07
NFKBIA	transcription regulator	-2.1	1.44E-06
STAT1	transcription regulator	-2.399	2.45E-06
IRF3	transcription regulator	-2.635	7.04E-06
IFN Beta	group	-3.082	7.76E-06
PKA	complex	-2.049	1.25E-05
IFNB1	cytokine	-2.65	1.78E-05
Jnk	group	-2.124	1.87E-05
RELA	transcription regulator	-2.614	1.96E-05
Ifnar	group	-2.593	2.06E-05
NFkB (complex)	complex	-2.438	5.56E-05
Creb	group	-2.363	1.72E-04
IFNA2	cytokine	-2.579	6.18E-04

#### 3.1.2 Comparison of gene expression in murine *Mirc11^-/-^ and Mir146a^-/-^* hematopoietic progenitors

*Mirc11^-/-^* murine HSPC gene expression inversely contrasts with the phenotype of *mmu-MiR146^-/-^* HSPCs, which have increased inflammatory signaling (26, 39). *Mir146a^-/-^* mice have low grade chronic inflammation and with age acquire myeloproliferative disorders, myeloid malignancies, and/or bone marrow failure. As opposed to the B lymphopoiesis at the expense of myelopoiesis observed in *Mirc11^-/-^* mice, *Mir146a^-/-^* bone marrow hematopoiesis skews toward myelopoiesis (39). Interestingly, activation of p65/NF-κB by bacterial lipopolysaccharide (LPS) exerts opposing effects on miRNA expression, up-regulating *Mir146a* while repressing *Mirc11* (35, 48, 49). Comparing RNA-seq data of *Mir146a^-/-^* [GSE87453] (50) and *Mirc11^-/-^* [GSE160215] (41) murine hematopoietic progenitors revealed opposing effects on inflammatory gene signatures. Pathway analysis revealed that *Mirc11^-/-^* has the opposite effect on TLR and Interferon signaling compared to *Mir146a^-/-^* (**Table 2**) (50).

**Table 2:** MIRC11 and miR-146a oppositely regulate inflammatory pathways in myeloid progenitor cells.

Gene set library	Pathway	z-score Mirc11-/-	-log(p-val) Mirc11-/-	z-score Mir146a-/-	-log(p-val) Mir146a-/-
Ingenuity Canonical Pathways	IL-8 Signaling	-2.12	1.64	2.14	2.02
Ingenuity Canonical Pathways	TREM1 Signaling	-1.34	1.69	1.67	2.98
KEGG Pathways	Sphingolipid metabolism Homo sapiens hsa00600	-1.01	2.97	5.11	2.97
KEGG Pathways	Toll-like receptor signaling pathway Homo sapiens hsa04620	-1.55	4.16	3.40	2.86
KEGG Pathways	NF-kappa B signaling pathway Homo sapiens hsa04064	-1.60	4.69	3.44	2.64
Reactome Pathways	Interferon alpha/beta signaling_Homo sapiens_R-HSA-909733	-0.40	1.56	2.86	3.36
Reactome Pathways	Interferon gamma signaling_Homo sapiens_R-HSA-877300	-0.50	1.54	3.44	2.64
Reactome Pathways	Interferon Signaling_Homo sapiens_R-HSA-913531	-1.19	1.98	3.53	2.15
WikiPathways	Toll-like Receptor Signaling Pathway_Homo sapiens_WP75	-1.98	4.31	3.53	2.98
WikiPathways	Type II interferon signaling (IFNG)_Mus musculus_WP1253	-1.33	3.91	6.25	2.96
WikiPathways	Type II interferon signaling (IFNG)_Homo sapiens_WP619	-0.61	2.67	5.41	2.67
WikiPathways	Regulation of toll-like receptor signaling pathway Homo sapiens_WP1449	-2.10	4.91	2.55	2.04

### 3.2 Characterization of bone marrow hematopoiesis in mice lacking both MIRC11 and miR-146a

#### 3.2.1 *Mir146a* deletion does not significantly affect *Mirc11^-/-^* myelopoiesis in young mice

**Figure 2:**
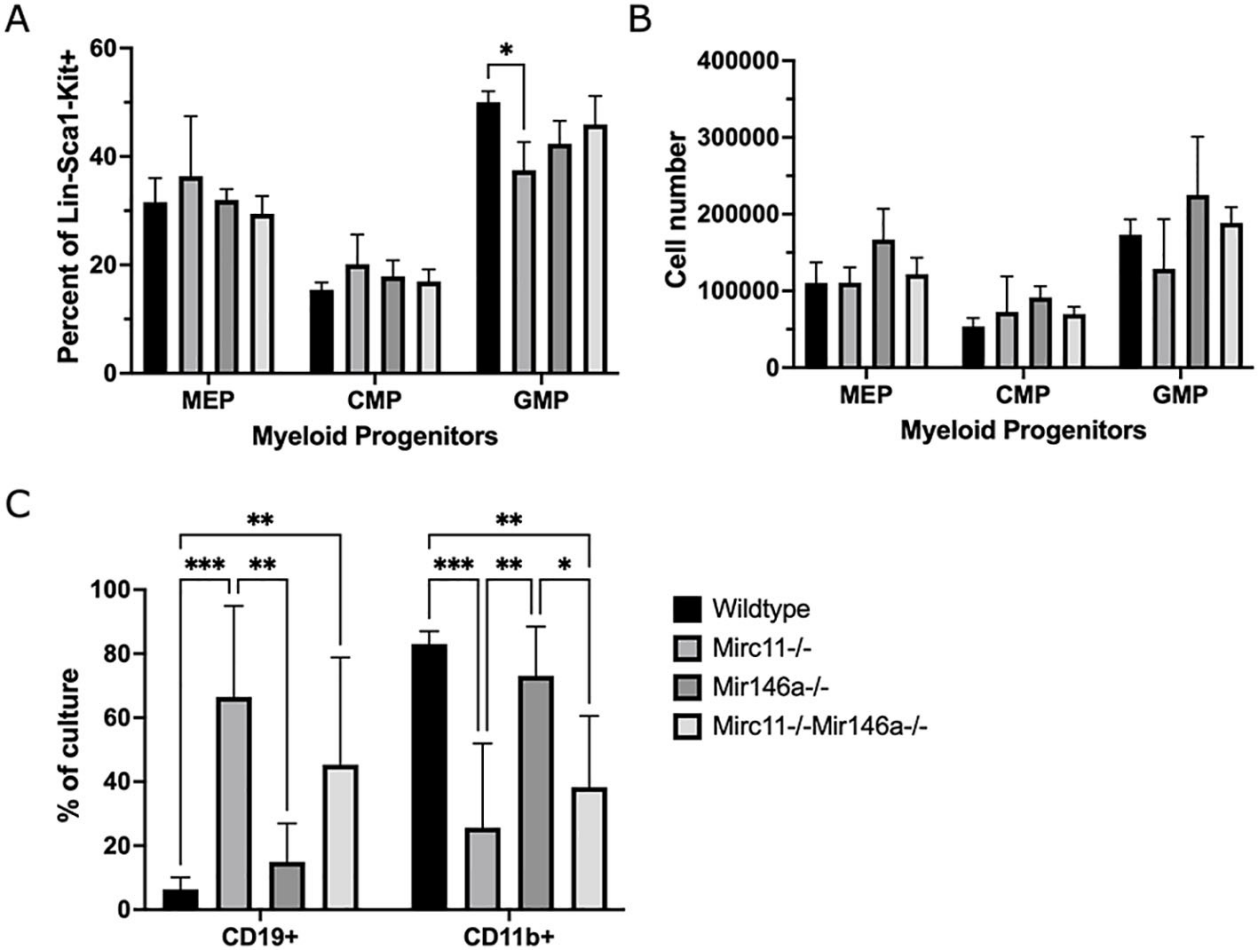
Analysis of hematopoiesis in young wildtype, *Mirc11^-/-^*, *Mir146a^-/-^* and double knockout mice. Nucleated bone marrow cells collected from 8-week-old mice were analyzed by flow cytometry for expression of cell surface markers or used for HSPC differentiation on OP9 stromal cells. **(A, B)** Myeloid progenitor populations were identified in the bone marrow by flow cytometry. The percent **(A)** of Lin-Sca1-cKit+ and the total number **(B)** of progenitor is shown **(C)** Lineage-negative (Lin-) progenitors were cultured on OP9 cells in the presence of Flt3L and IL-7 for 15 days. Differentiation to the myeloid or B-cell lineages was evaluated by cell surface of expression of CD11b and B220 (representative plots shown). Results are averages of 4–6 independent cultures for each genotype. Bone marrow obtained from a single mouse was used for each culture. Error bars denote standard deviation. *(p<0.05), **(p<0.01), ***(p<0.001), ****(p<0.0001).

Given the opposing roles of mmu-MIRC11 and mmu-miR-146a on hematopoietic inflammatory signaling, we crossed *Mirc11^-/-^* and *MiR146a^-/-^* mice to determine the extent to which they oppose each other’s function. Double knockout mice were viable and fertile. We examined bone marrow hematopoiesis in wildtype, *Mirc11^-/-^, Mir146a^-/-^ and Mirc11^-/-^Mir146a^-/-^* mice starting at 8 weeks up to 12 months of age. Consistent with prior findings, we observed a significant decrease in the percentage of bone marrow granulocyte-monocyte progenitors (GMPs) between wildtype and *Mirc11^-/-^* mice at 8 weeks of age (**Figure 2A**; **Supplementary Figure 1A**) (33). The same trend was seen in total numbers of GMPs though this was not statistically significant (**Figure 2B**). In contrast there was no significant difference in the bone marrow percentage of GMPs between wildtype and *Mirc11^-/-^Mir146a^-/-^* mice. However, although on average the percentage of bone marrow GMPs was increased in double knockout mice compared to *Mirc11^-/-^*, it did not rise to a level of significance. To determine if compound loss of these miRNAs impacted myeloid and lymphoid differentiation, we performed OP9 co-culture assays with Lin-Sca1+cKit+ (LSK) HSPCs. We observed significantly increased B lymphopoiesis (CD19+ cells) and decreased myelopoiesis (CD11b+ cells) in *Mirc11^-/-^* cultures compared to wildtype (**Figure 2C**, **Supplementary Figure 1B**). *Mirc11^-/-^Mir146a^-/-^* cells had significant increases in CD19+ and decreases in CD11b+ populations compared to wildtype however the differences on average were not as substantial as between wildtype and the single *Mirc11* mutant cells. However, despite the trend, there was not a significant difference in the CD19+ and CD11b populations comparing *Mirc11^-/-^* and *Mirc11^-/-^Mir146a^-/-^* cultures. Examination of hematopoiesis in 8-week-old mice did not reveal any significant rescue of the previous described hematopoietic alterations observed in *Mirc11^-/-^* mice when *Mir146a* is deleted. However, on average we did observe that the percentage of myeloid cells increased in double knockout mice compared to *Mirc11^-/-^* so a modifying effect of miR-146a loss cannot be completely ruled out.

#### 3.2.2 *Mirc11* loss does not rescue *Mir146a^-/-^* bone marrow failure

**Figure 3:**
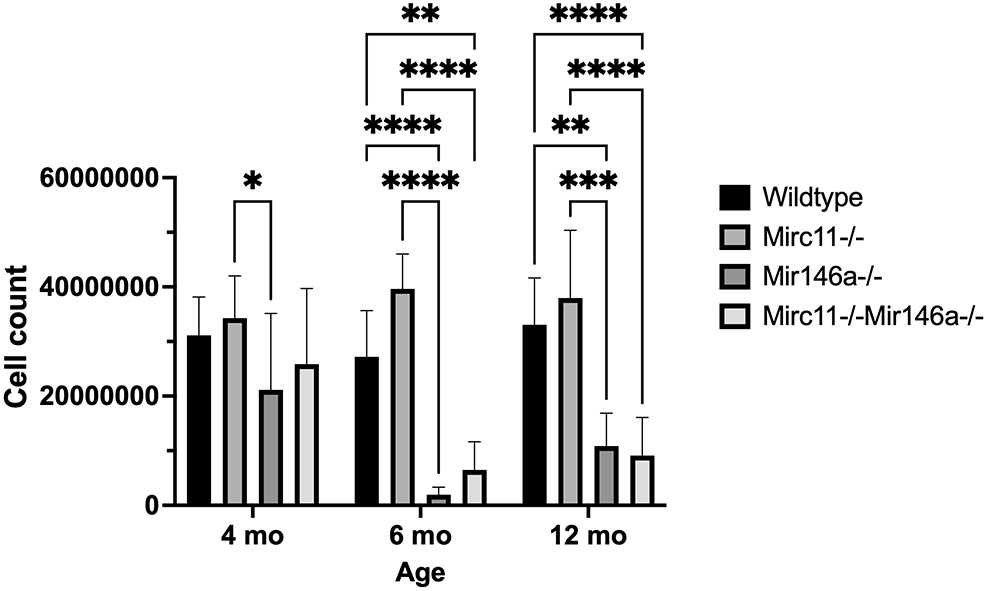
Analysis of bone marrow isolated from aged wildtype, *Mirc11^-/-^*, *Mir146a^-/-^* and double knockout mice. Bone marrow isolated from mice aged 4, 6, and 12 months. Average total number of cells isolated from each genotype. Error bars denote standard deviation. *(p<0.05), **(p<0.01), ***(p<0.001), ****(p<0.0001). At least 4 mice were examined for each genotype and timepoint.

Examining the *Mirc11*/*Mir146a* mutant mice as they aged, we observed decreased bone marrow cellularity in *Mir146a^-/-^* and *Mirc11^-/-^Mir146a^-/-^* mice as early as 4 months of age which persisted and was significant at 6 and 12 months of age (**Figure 3**). Bone marrow cell numbers at 6 months were higher in double knockout mice compare to *Mir146^-/-^* but the cellularity of both were dramatically less compared to wildtype animals. Examining bone marrow Lin-Sca1+cKit+ HSPCs (LSK) population at 4 months of age we observed that double knockout LSKs were significantly increased compared to the other 3 genotypes. The percentage of double knockout LSK cells was increase compared to wildtype and *mmu-Mirc11^-/-^* but was like what was observed in *Mir146a^-/-^* mice (**Supplementary Figure 2**). At 6 months, *mmu-MiR146a* mutant mice had less LSK cells compared to wildtype and double knockout mice however the frequency of LSKs remained higher compared to wildtype but was similar to what was observed in double knockout mice. Unfortunately, due to technical problems we were unable to collect LSK data from year old mice.

#### 3.2.3 MIRC11 loss does not significantly affect, myeloid progenitor reduction during *MiR146a^-/-^* bone marrow failure

**Figure 4:**
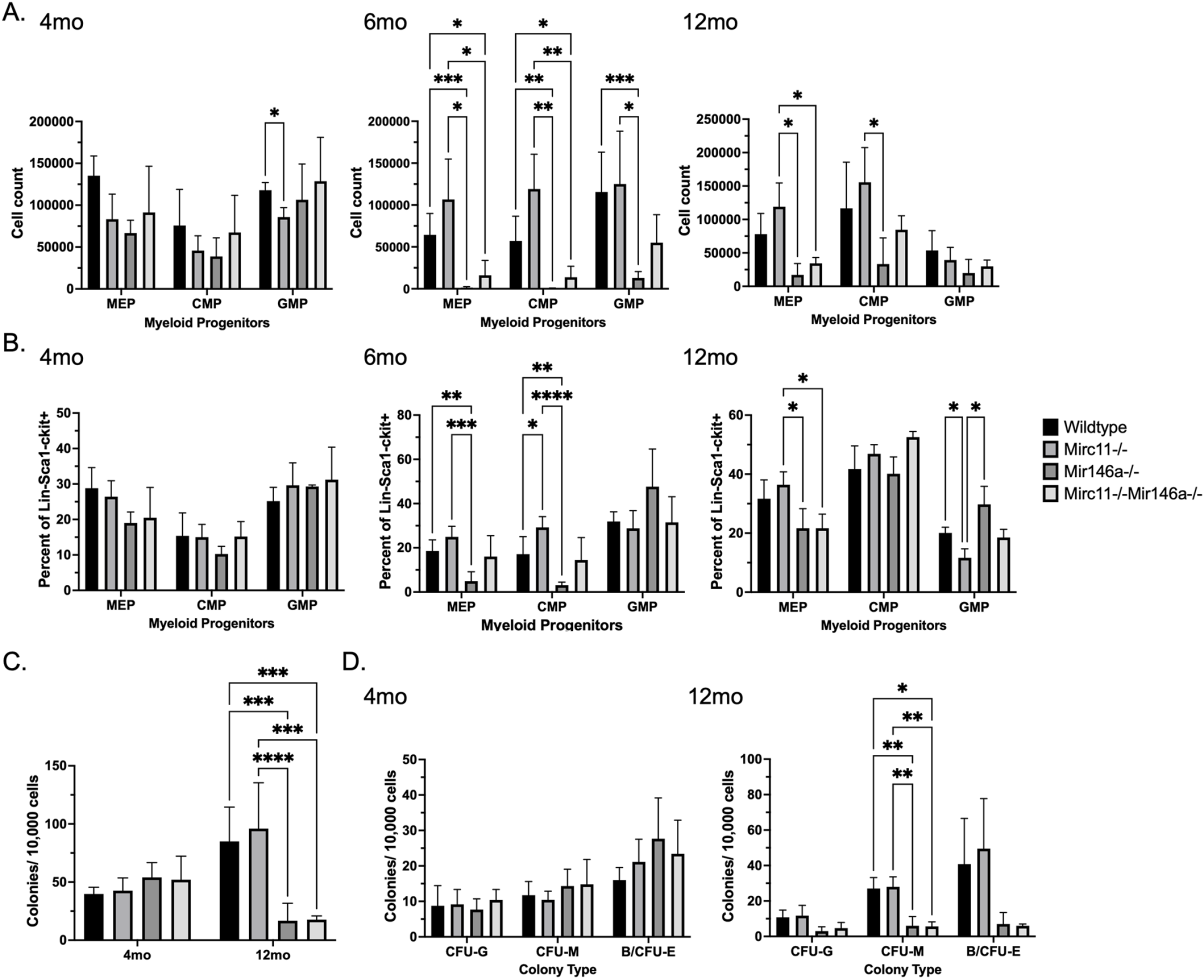
Analysis of changing myeloid progenitors in the bone marrow of wildtype, *Mirc11^-/-^, Mir146a^-/-^* and double knockout mice over time. Bone marrow from mice with indicated genotypes was isolated from tibias and femurs at the indicated ages. Bone marrow myeloid progenitors, **(A)** Total number, **(B)** Percent of bone marrow. 10,000 bone marrow cells were plated for hematopoietic colony assays. C.Total number of hematopoietic colonies. **(D)** Total number of myeloid colonies. Error bars denote standard deviation. *(p<0.05), **(p<0.01), ***(p<0.001), ****(p<0.0001). 3–7 mice were examined for each genotype and timepoint except for myeloid progenitors 4mo *Mir146a^-/-^* (total and %) where 2 mice were assayed. CFU, colony forming unit; BFU, burst forming unit; B/CFU-E, erythroid progenitor colonies; CFU-M, monocytic progenitor colonies, CFU-G, granulocytic progenitor colonies.

Examining bone marrow myeloid progenitor populations at 4 months of age, we observed no differences in common myeloid progenitor (CMP), megakaryocyte/erythroid progenitor (MEP), and GMP numbers or percentages between wildtype and *Mir146a^-/-^* mice (**Figure 4**; **Supplementary Figure 3**). There was a decrease in GMP number between 4-month-old wildtype and *Mirc11^-/-^* mice (33) (**Figure 4A**). There also was a decrease in the percentage of GMPs in *Mirc11^-/-^* bone marrow in 12-month-old mice but this was not observed at other timepoints (**Figure 4B**). In line with the bone marrow failure of *Mir146a^-/-^* mice, myeloid progenitors decreased over time in *Mir146a^-/-^* and *Mirc11^-/-^MiR146a^-/-^* mice compared to wildtype and *Mirc11^-/-^* mice (**Figures 4A, B**). At 6 months we observed a decrease in both total numbers and percentages of MEPs and CMPs in miR-146a mutants compared to wildtype mice (**Figures 4A, B**). While on average, *Mirc11^-/-^Mir146a^-/-^* mice had increased MEPs and CMPs at 6 and 12 months compared to *Mir146a^-/-^* mice, with increased percentages observed at 6 months, we did not observe these differences to be significant. If there is an effect of MIRC11 loss on the altered production of hematopoietic progenitors in *Mir146a^-/-^* mice, it is very modest.

To further characterize the bone marrow hematopoietic progenitor content, we performed hematopoietic colony assays (HCAs) (45). Consistent with flow cytometric analysis we observed no significant differences in progenitor numbers in HCAs at 4 months (**Figures 4C, D**). At 12 months there was an equivalent decrease in monocytic (CFU-M) colonies in *Mir146a^-/-^* and *Mirc11^-/-^Mir146a^-/-^* assays compared to wildtype. There was a similar trend in decreased erythroid BFU-E/CFU-E and granulocytic CFU-G in *Mir146a^-/-^* and *Mirc11^-/-^Mir146a^-/-^* cultures compared to wildtype. Multipotential colonies CFU-GEMM (Generated primarily from CMPs (Common Myeloid Progenitors) and MPPs (Multipotent Progenitors) as well as HSCs), CFU-GM (Generated from GMPS), and CFU-EMeg (Generated from MEPs) along with the unipotent colony megakaryocytic CFU-Meg were scored. These counts were included in total counts however for each genotype the number of CFU-GEMM, -GM, -EMeg and -Meg varied from 0–2 colonies which did not allow us to determine statistical differences between genotypes. The hematopoietic colony assays did not reveal any significant changes between *Mir146a^-/-^* and *Mirc11^-/-^Mir146a^-/-^* mice in the generation of hematopoietic progenitor colonies. The production of progenitors appeared to be equally perturbed in both strains.

#### 3.2.4 Mature hematopoietic *Mirc11^-/-^MiR146a^-/-^* bone marrow populations resemble *MiR146a^-/-^* bone marrow

**Figure 5:**
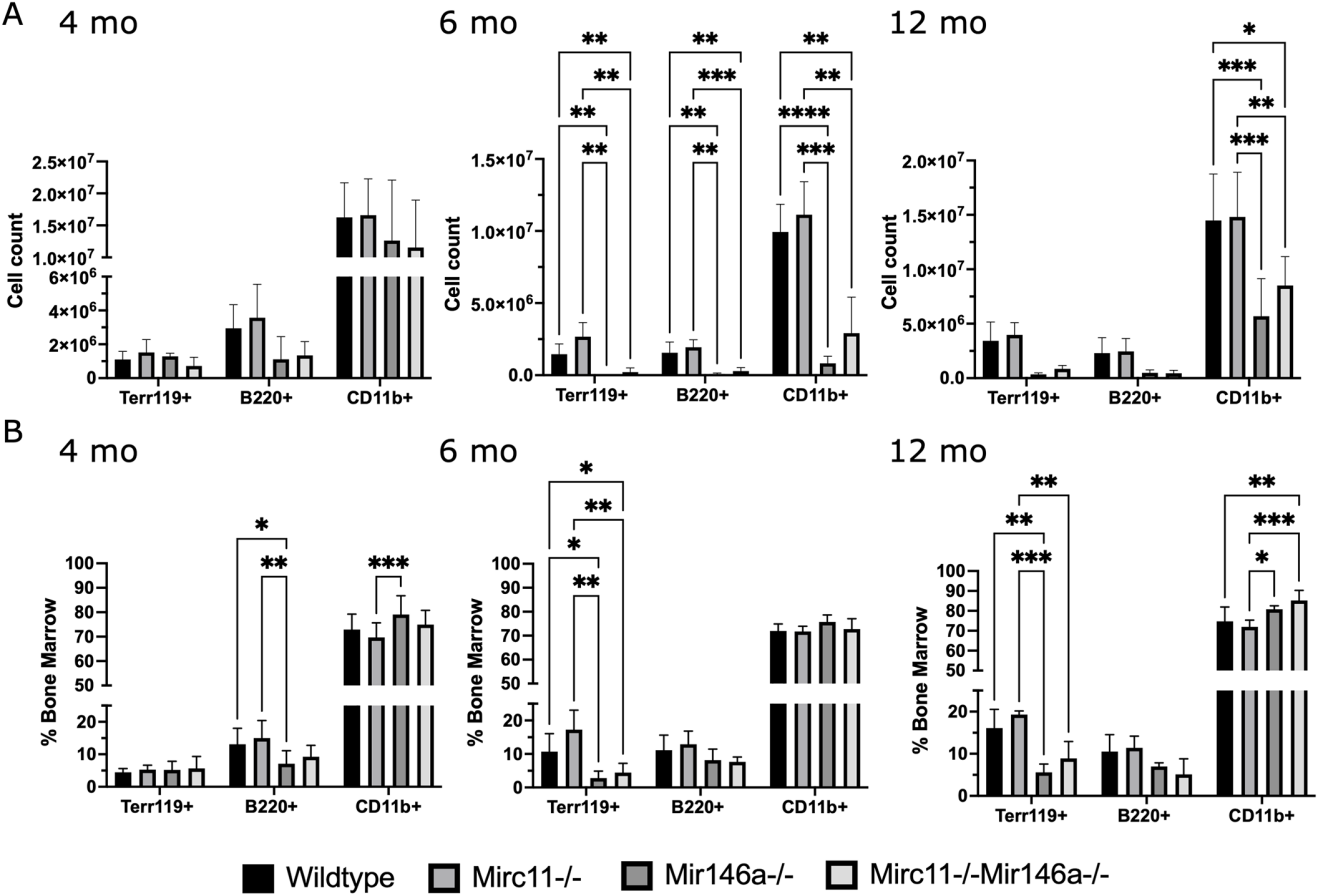
Flow cytometric analysis of bone marrow mature hematopoietic populations. Bone marrow from mice with indicated genotypes was isolated from tibias and femurs of mice aged 4, 6, and 12 months. Cells were stained with fluorescently tagged antibodies recognizing TERR119 (erythroid), CD11b (myeloid), and B220+ (B cells). **(A)** Total number of each population in the bone marrow. **(B)** Percentage of each lineage contributing to the bone marrow. *(p<0.05), **(p<0.01), ***(p<0.001), ****(p<0.0001). Error bars denote standard deviation. 3–11 mice were examined for each genotype and timepoint.

We next investigated the impact of compound miRNA loss on mature cell populations. Due to decreased bone marrow cellularity, the total number of Terr119+ (erythroid), B220+ (B cell), and CD11b+ (myeloid) cells significantly decreased at 6 and 12 months of age in *Mir146a^-/-^* and *Mirc11^-/-^MiR146a^-/-^* animals (**Figure 5A**). At 4 months of age there was a decrease in the percentage of B220+ B cells in *mmu-Mir146a^-/-^* bone marrow compared to wildtype (**Figure 5B**; **Supplementary Figure 4**). 6- and 12- month- old *Mir146a^-/-^* mice had decreased percentages of erythroid cells (**Figure 5B**; **Supplementary Figure 5**). In both cases (B cells and erythroid), we did not observe a significant difference in percentages between miR-146a deficient mice and double deficient mice.

### 3.3 Analysis of gene expression induced by TLR ligands in *Mirc11^-/-^* and *Mir146a^-/-^* macrophages

#### 3.3.1 Characterization of TLR responses in *Mirc11^-/-^Mir146a^-/-^* macrophages

**Figure 6:**
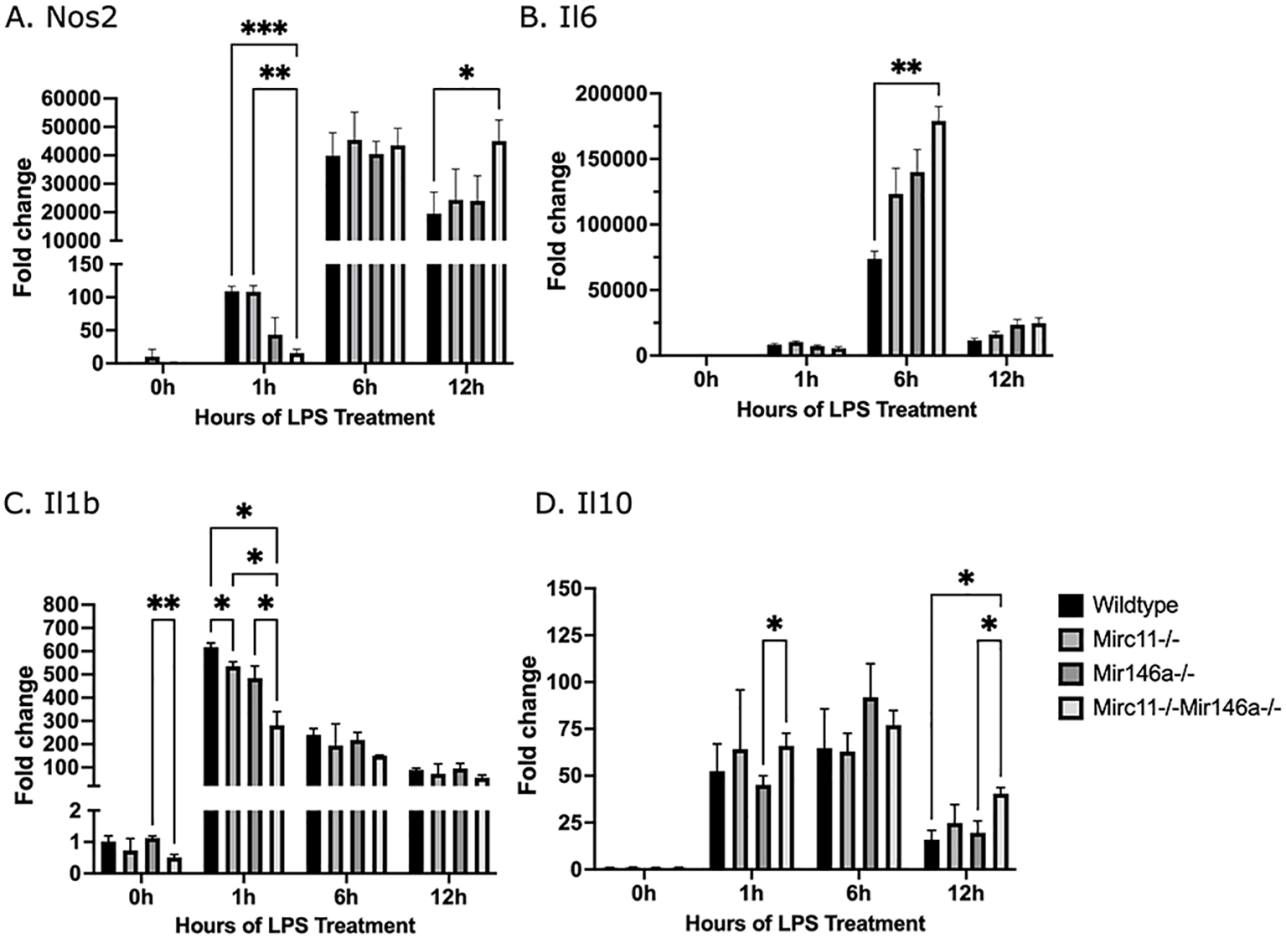
Inflammatory gene expression in bone marrow derived macrophages treated with Lipopolysaccharide. Macrophages were prepared from bone marrow isolated from indicated genotypes at 6–8 weeks of age. Cells were treated with 100ng/ml LPS for the indicated times and RNA collected for RT-PCR analysis. BMDM were treated LPS for 0, 1, 6, and 12h and assayed for expression of **(A)** *Nos2*, **(B)** *Il6* **(C)** Il6 and **(D)** Il10. Error bars denote standard deviation. *(p<0.05), **(p<0.01), ***(p<0.001). BMDM derived from 3 independent mice for each genotype were used for each assay.

Given mmu-MIRC11 and mmu-miR-146a deficient hematopoietic progenitors inversely regulated TLR activation of NF-κBsignaling (**Tables 1**, **2**) we next sought to determine if this functionally persisted in mature myeloid cells. NF-κB regulated gene expression was assayed in bone marrow derived macrophages (BMDMs) in response to TLR ligand, lipopolysaccharide (LPS/TLR4 ligand). BMDM lacking mmu-miR-146a have increased expression of NF-κB activated inflammatory genes *Nos2*, *Il1b* and *Il6* in response to LPS treatment (39, 51) whereas LPS treatment of *Mirc11^-/-^* BMDMs results in increased expression of the anti-inflammatory gene and miR-27a (MIRC11 miRNA) target *Il10* (41, 52). Interestingly at the timepoints we examined we did not observe increased *Nos2* expression in *Mir146a^-/-^* BMDMs compared to wildtype. At 1h we observed a decrease in *Nos2* in double knockout macrophages compared to wildtype and *Mirc11^-/-^* cells (**Figure 6A**). Interestingly, highest expression of *Nos2* was observed after 12h of LPS treatment of the double knockout cells. Similarly, the highest expression of *Il6* was observed in the double knockout cells (**Figure 6B**). When we examined *Il1b* expression we observed a similar synergy but in the opposite direction (**Figure 6C**). As observed previously (41), *mmu*-*Mirc11^-/-^* cells had decreased expression of pro-inflammatory cytokine *Il1b* 1h post-LPS treatment however the greatest decrease was observed in *Mirc11^-/-^Mir146a^-/-^* BMDMs. Lastly, the expression of Il10, which is a well characterized target of MIRC11 miRNA, miR-27a (52), was significantly increased in double knockout cells at 12h post LPS treatment compared to wildtype and *Mir146a^-/-^* macrophages (**Figure 6D**).

In addition, we examined *Nos2* and *Il10* expression after treatment of BMDMs with two other TLR agonists, pIpC (TLR2 ligand) and Pam_3_CSK_4_ (activates TLR2/TLR1 heterodimer). Pam_3_CSK_4_ treatment showed a similar trend for *Il10* that was seen with LPS treatment where highest expression was observed in double knockout cells. However, Nos2 expression on average was highest in *mmu-MiR146a^-/-^* cells (**Supplementary Figures 6A, B**). Treatment of macrophages with pIpC (**Supplementary Figures 6C, D**) in contrast to results observed with LPS and Pam_3_CSK_4_ showed the *Mirc11^-/-^* phenotype dominant in the expression of *Il10* and *Nos2*. Expression of *Nos2* appeared to be equivalently reduced compared to wildtype in both *Mirc11^-/-^* and *Mirc11^-/-^Mir146a^-/-^* BMDM cultures. Similarly, *Il10* was equivalently induced in the *mmu*-*Mirc11* and double mutant cells. However, the trends we observed with the TLR ligand Pam_3_CSK_4_ and pIpC were not considered significant except for the decrease in *Il10* expression observed at 24h post-treatment in *Mir146a^-/-^* cells. In these experiments we did not observe the expected *Il10* increase in single mutant *Mirc11^-/-^* cells.

The results of the TLR ligand stimulation do not support a hypothesis in that MIRC11 and miR-146a miRNAs oppose each other’s regulation of TLR signaling. Interestingly their activities seem gene-specific, and they can synergize to repress or activate a TLR stimulated gene.

#### 3.3.2 MIRC11 miRNAs and miR-146a regulate each other’s gene expression in bone marrow derived macrophages

**Figure 7:**
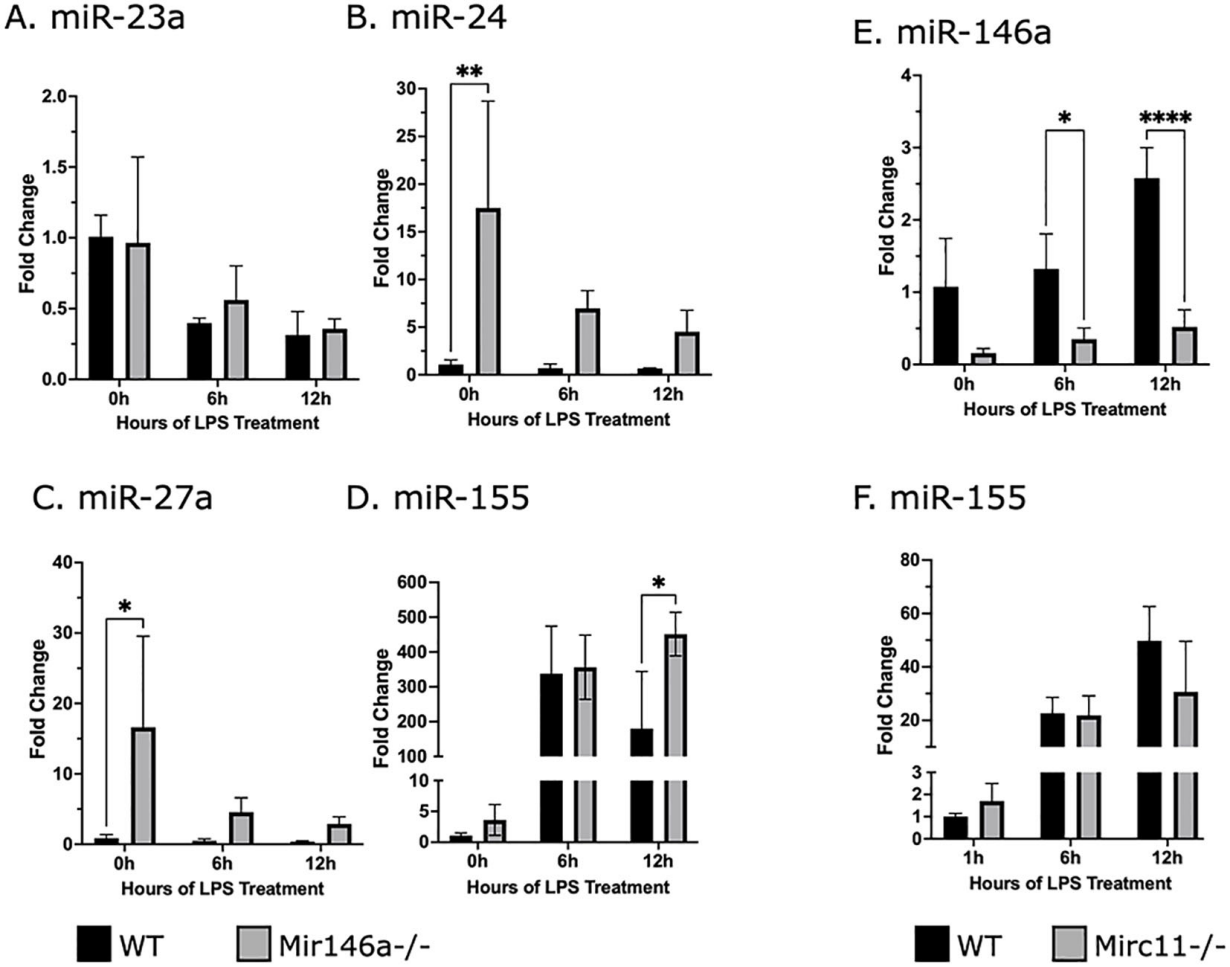
MiRNA expression in LPS treated wildtype, *Mirc11^-/-^* and *Mir146a^-/-^* BMDMs. Macrophages were incubated with 100 ng/ml LPS for 0, 6 and 12h and harvested for RNA. **(A)** Wildtype and *Mir146a^-/-^* cells were assayed for the expression of miRNAs A) mmu-miR-23a, **(B)** mmu-miR-24, **(C)** mmu-miR-27a, and **(D)** mmu-miR-155. Wildtype and *Mirc11^-/-^* cells were assayed for expression of **(E)** mmu-miR-146a and **(F)** mmu-miR-155. Error bars denote standard deviation. *(p<0.05), **(p<0.01), ***(p<0.001), ****(p<0.0001). BMDM derived from 3 independent mice for each genotype were used for each assay.

In response to LPS challenge, MIRC11 miRNA expression is repressed whereas miR-146a expression is induced (35, 48, 49). To determine how these two miRNA genes respond to each other’s loss during acute inflammation, we examined expression of mmu-MIRC11 miRNAs and mmu-miR-146a in single knockout BMDMs treated with LPS for 0, 6 and 12 hours (**Figures 7A–D**). In *Mir146a^-/-^* BMDMs, mmu-miR-23a expression was unchanged. Strikingly, both mmu-miR-27a and mmu-miR-24–2 exhibited 15-fold increased expression in *Mir146a^-/-^* BMDMs compared to wildtype at baseline. Although mmu-miR-27a and mmu-miR-24–2 expression decreased at 6 and 12 hours post-LPS challenge in *Mir146a^-/-^* BMDMs compared to baseline, their expression was around 5-fold higher compared to wildtype BMDMs. We also interrogated the expression of pro-inflammatory miRNA mmu-miR-155 (53). We observed significant increase of mmu-miR-155 at 12 hours post LPS treatment in *Mir146a^-/-^* BMDMs, but no difference 6h. Examining mmu-miR-146a expression in *Mirc11^-/-^* BMDM revealed that mmu-miR-146a was significantly decreased in *Mirc11^-/-^* cells at all timepoints examined (**Figures 7E, F**). Mmu-miR-155 expression was unchanged in *Mirc11^-/-^* BMDMs upon LPS challenge. The data demonstrates that MIRC11 and miR-146a can affect each other’s expression.

## 4 Discussion

Inflammatory signaling plays a central role in regulating HSPC function, influencing lineage commitment, stem cell maintenance, and immune responsiveness (3, 54). However, maintaining appropriate levels of inflammatory signaling is essential, as both insufficient and excessive activation disrupt hematopoietic homeostasis and contribute to hematologic disease. In this study, we identify the microRNA cluster MIRC11 as a positive regulator of inflammatory signaling in hematopoietic cells and demonstrate that its activity contrasts with the anti-inflammatory function of miR-146a, a well-established negative regulator of NF-κB signaling. MIRC11 miRNAs are reported to enhance NF-κB activity through the repression of negative NF-κB regulators including A20, CYLD, CBL-B and ITCH (36, 37). Our findings reveal that these miRNA pathways influence overlapping inflammatory networks but do not simply function as reciprocal regulators of hematopoiesis. Instead, genetic and functional analyses indicate that mmu-MIRC11 and mmu-miR-146a contribute to the fine-tuning of inflammatory signaling thresholds that govern hematopoietic differentiation and immune activation. These results provide new insight into the microRNA-mediated regulatory circuits that help maintain inflammatory balance in hematopoietic tissues. Specifically, our results show that loss of mmu-MIRC11 reduces inflammatory signaling in hematopoietic progenitors, that mmu-MIRC11 miRNAs and mmu-miR-146a exert opposing effects on inflammatory pathway activity, and that combined disruption of these miRNAs produces modest hematopoietic interactions but complex, gene-specific effects on macrophage inflammatory responses.

Gene expression analysis of *Mirc11*-deficient murine hematopoietic stem and progenitor cells revealed reduced activation of multiple inflammatory pathways, including TNF, interferon, and TLR signaling networks (**Figure 1**; **Table 1**). These findings are consistent with the hematopoietic phenotype observed in MIRC11-deficient mice, which display decreased myeloid differentiation and increased lymphoid output (33). Inflammatory signaling is known to promote myeloid lineage commitment in HSPCs, and therefore reduced inflammatory pathway activity likely contributes to the altered lineage balance observed in *Mirc11^-/-^* mice (3). These results support the concept that mmu-MIRC11 miRNAs act to promote inflammatory signaling in hematopoietic progenitors and thereby influence hematopoietic lineage decisions.

In contrast, miR-146a is widely recognized as a negative regulator of inflammatory signaling. Previous studies have shown that miR-146a-deficient mice develop chronic inflammation and progressive hematopoietic defects characterized by impaired HSC function, cytopenias, and eventual bone marrow failure (26, 39, 40). Consistent with these observations, gene expression analyses of *mmu*-*Mir146a^-/-^* progenitors demonstrate increased activation of NF-κB–dependent inflammatory pathways. Interestingly, comparison of *mmu*-*Mirc11^-/-^* and *mmu*-*Mir146a^-/-^* transcriptional profiles revealed that several inflammatory signaling pathways are regulated in opposite directions by these miRNAs, suggesting that they may act as counterbalancing regulators of inflammatory signaling in hematopoietic cells (**Table 2**).

**Figure 8:**
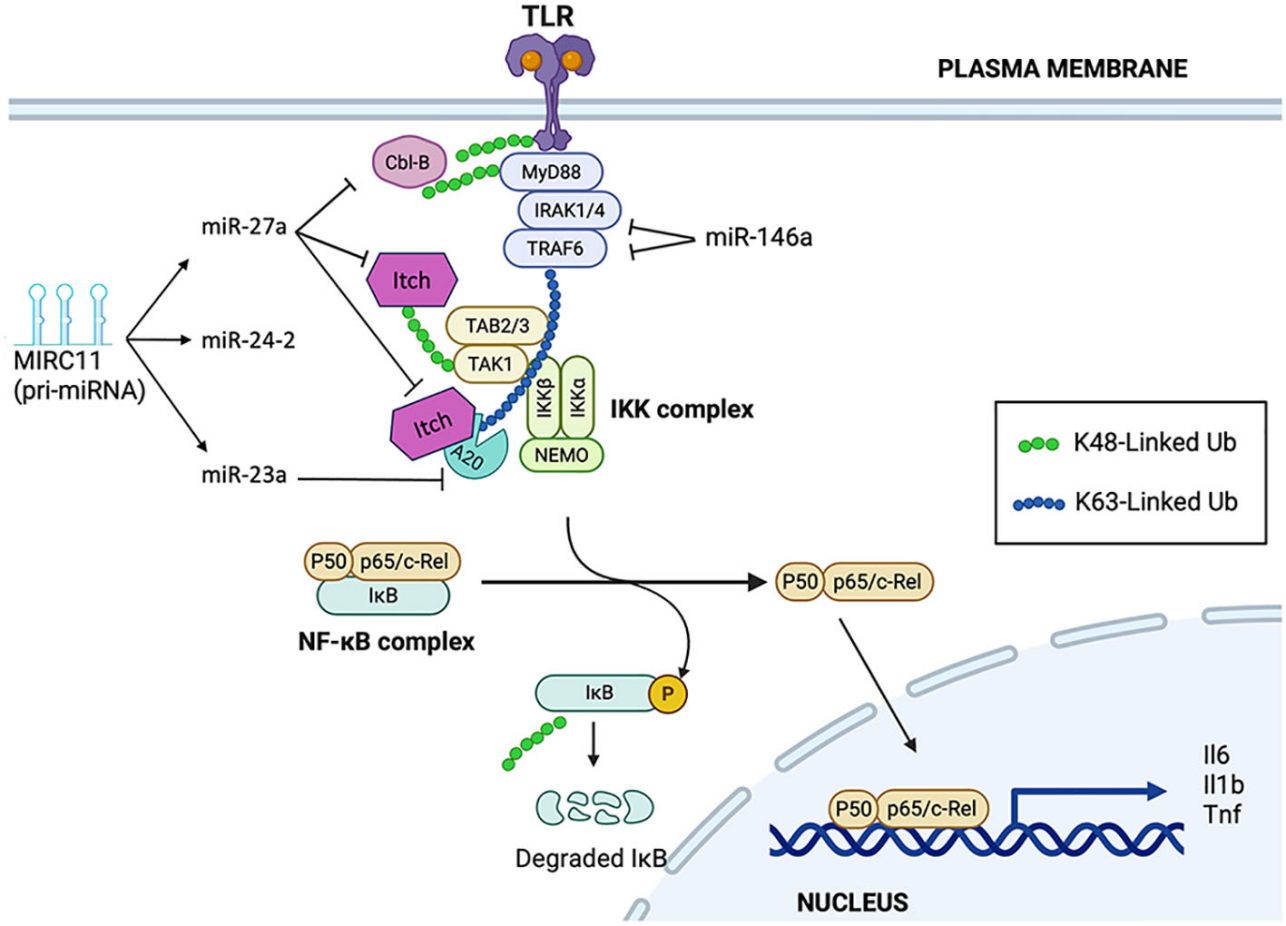
Opposing regulation of Toll like receptor signaling by MIRC11 miRNAs and miR-146a. MIRC11 miRNAs can increase TLR signaling and downstream activation through targeting E3 K48 ubiquitin ligases and K63 deubiquitinases. Targeting of ubiquitin ligases Cbl-B, and Itch by miR-27a and deubiquitinase A20 by miR-23a leads to stabilization of TLR, MyD88, TAK1, and TRAF6. Decreased A20 and Itch lead to stable K63 ubiquitinated TRAF6 which enhances TAK1 activation of IKK complex and downstream activation of NF-κB. Mir-146a expression opposes activation of NF-kB by TLR through decreases protein levels of the IRAK1 and TRAF6 leading to reduced IKK activation and decreased inhibition of NF-κB. Ub, Ubiquitin. Created with https://www.BioRender.com.

Mechanistically, the opposing effects of the MIRC11 miRNAs and miR-146a on inflammatory signaling arise from their distinct regulatory targets within TLR, TNFR, and IL-1R signaling pathways that lead to NF-κB activation (**Figure 8**). The MIRC11 cluster members miR-27a and miR-23a promote NF-κB activation by suppressing key negative regulators of signaling. MiR-27a inhibits the ubiquitin ligases CBL-B and ITCH, which normally dampen NF-κB signaling by catalyzing K48-linked ubiquitination and degradation of signaling intermediates such as MyD88, TAB1, and TRAF6 (36, 55, 56). Additionally, miR-27a directly targets TAB1 (57). In parallel, miR-23a represses expression of the deubiquitinase A20, which removes activating K63-linked ubiquitin chains from TRAF6 (37, 58). Because K63-linked ubiquitination of TRAF6 is required for assembly of signaling complexes that activate TAK1 and downstream NF-κB, repression of CBL-B, ITCH, and A20 by MIRC11 miRNAs enhances inflammatory signaling downstream of TLRs, TNFRs, and IL-1R. In contrast, miR-146a acts as a negative regulator of NF-κB signaling by directly targeting TRAF6 and IRAK1 (59, 60), thereby limiting activation downstream of TLR and IL-1 receptor pathways. Thus, whereas miR-146a restrains inflammatory NF-κB activation, MIRC11 miRNAs amplify and sustain it.

Based on these opposing regulatory activities, we hypothesized that MIRC11 and miR-146a might function together to buffer NF-kB and other inflammatory signaling pathways in hematopoietic cells. To test this possibility, we generated *Mirc11^^-^/^-^^Mir146a^^-^/^-^^* double knockout mice and examined their hematopoietic phenotypes. In young mice, there was a trend that loss of miR-146a would increase the bone marrow myeloid population that is decreased in *Mirc11*-deficient animals, (**Figure 2**). However, the increase did not rise to the level of statistical significance. Even if there is a modest increase in myelopoiesis mediated by loss of miR-146a expression, it is clear that the overall hematopoietic phenotype of double knockout mice was not intermediate between the two single mutants. Longitudinal analysis revealed that double knockout animals ultimately developed hematopoietic defects similar to those observed in *Mir146a^^-^/^-^^* mice, including progressive loss of bone marrow cellularity and reduced hematopoietic progenitor numbers (**Figures 3**, **4**; **Supplementary Figure 2**). These findings indicate that mmu-MIRC11 deficiency is unable to prevent the chronic inflammatory pathology associated with mmu-miR-146a loss. Interestingly as *Mirc11^-/-^* mice age we did not observe the pronounced decrease in myelopoiesis and enhanced B lymphopoiesis we observed in 6–8 week old mice. This may be due to compensatory mechanisms in the bone marrow microenvironment that are activated to restore normal hematopoietic homeostasis. The most dramatic effects on myeloid-lymphoid differentiation have been observed with OP9/*mmu*-*Mirc11^-/-^* HSPC co-cultures where the environment is static and user defined.

Analysis of inflammatory gene expression in macrophages further revealed complex interactions between these miRNA pathways. We initially hypothesized that inflammatory responses in double knockout macrophages would be intermediate between those observed in the single mutants. Instead, our results demonstrated that deletion of both *mmu*-*Mirc11* and *mmu*-*Mir146a* frequently amplified the transcriptional phenotype associated with individual mutations (**Figure 6**; **Supplementary Figure 6**). For example, pro-inflammatory genes that were elevated in *Mir146a*-deficient macrophages were further increased in double knockout cells following LPS stimulation, whereas genes reduced in *Mirc11*-deficient cells were further suppressed in the double mutant background. These findings suggest that interactions between these miRNAs cannot be explained by a simple antagonistic relationship.

One possible explanation for this observation is that MIRC11 and miR-146a regulate overlapping but distinct networks of inflammatory targets. The combined loss of both regulatory pathways may therefore destabilize inflammatory signaling networks rather than restore them to an intermediate state. In addition, our data demonstrate that these miRNAs can influence each other’s expression in macrophages, suggesting that additional feedback mechanisms contribute to their regulatory interactions during inflammatory responses (**Figure 7**).

Taken together, our results highlight the complexity of miRNA-mediated regulation of inflammatory signaling in hematopoietic cells. While MIRC11 and miR-146a exert opposing effects on inflammatory pathway activation, their combined genetic disruption does not simply normalize inflammatory signaling. Instead, the interaction between these pathways appears to depend on the specific cellular context and the downstream gene targets involved. From a broader perspective, these findings underscore the importance of microRNA networks in maintaining immune homeostasis. Inflammatory signaling must be precisely regulated in HSPCs to allow appropriate immune responses while avoiding the deleterious effects of chronic inflammation. Our data suggest that MIRC11 and miR-146a contribute to this regulatory balance but do so through complex and context-dependent mechanisms.

Finally, these findings raise the possibility that coordinated manipulation of miRNA pathways could be used to modulate immune responses therapeutically. Because MIRC11 promotes inflammatory signaling whereas miR-146a acts as a negative regulator, targeted modulation of these pathways may allow selective enhancement or suppression of specific inflammatory gene programs. However, our results also highlight the challenges of such approaches, as the combined manipulation of these miRNAs does not uniformly amplify or suppress inflammatory signaling. A deeper understanding of the regulatory networks controlled by these miRNAs will therefore be required before their therapeutic potential can be fully realized.

## Data Availability

RNA-seq data performed for this studying of RNA obtained from wildtype and MIRC11 HSPCs (Bone marrow Lin-Sca-1+c-Kit+) has been deposited at NCBI GEO (https://www.ncbi.nlm.nih.gov/geo/), accession number GSE335378. Previously reported sequencing data for *Mirc11^-/-^* and *Mir146a^-/-^* myeloid progenitors (bone marrow Lin-Sca-1-cKit+) is accessible by querying the accession numbers GSE160214 and GSE87453 respectively at NCBI GEO.

## Supplementary material

Supplementary Figure 1 Representative flow cytometric analysis of bone marrow cells isolated from 8-week-old mice.

Supplementary Figure 2 Flow cytometric analysis of bone marrow LSK cells.

Supplementary Figure 3 Representative Flow cytometric analysis of myeloid progenitor populations in bone marrow.

Supplementary Figure 4 Representative Flow cytometric analysis of mature myeloid and B cell populations.

Supplementary Figure 5 Representative Flow cytometric analysis of the Ter119+ erythroid cell population1.

Supplementary Figure 6 Inflammatory gene expression in bone marrow derived macrophages treated with Toll like receptor (TLR) ligands.

Supplementary Table 1 Differential expression of TNF, IL-6 and Il-1β regulated genes between wildtype and *Mirc11^-/-^* primary LSK cells determined by RNA-seq analysis.
