# Supplementary material for: Balancing inflammatory signaling in hematopoiesis: roles of MIRC11 and miR-146a
Source: Front Immunol. 2026 Jul 8;17:1828453. doi: 10.3389/fimmu.2026.1828453 (PMC13388049; doi:10.3389/fimmu.2026.1828453)
Supplement: Supplementary Figure 1 — Representative flow cytometric analysis of bone marrow cells isolated from 8-week-old mice. [file DataSheet1.pdf]

Supplementary Material

1 Supplemental Material and Methods

RNA Sequencing

Library Preparation. RNA-seq libraries were prepared at the University of Notre Dame Genomics and Bioinformatics Core Facility. Total RNA samples were diluted 5 times prior to analysis. Sample concentration was measured using Qubit RNA HS Assay Kit (PN: Q32855; Invitrogen, Carlsbad, CA, USA). Total RNA was evaluated with Agilent Bioanalyzer 2100 System and Agilent RNA 6000 Nano Kit (PN: 5067-1511; Agilent Technologies, Santa Clara, CA). Samples with an RNA Integrity Number (RIN) of 7 or higher were qualified for library preparation. Total RNA in-put was normalized to 150 ng. Polyadenylated RNA molecules were selected for using NEBNext Poly(A) mRNA Magnetic Isolation Module (PN: E7490S/L; New England BioLabs, Ipswich, MA). Enriched polyadenylated RNA was converted into an Illumina library using NEBNext Ultra II RNA Library Prep with Sample Purification Beads (PN: E7775S/L; New England BioLabs, Ipswich, MA) and barcoded with NEBNext Multiplex Oligos for Illumina (Index Primers Set 1) (PN: E7335S/L; New England BioLabs, Ipswich, MA) or NEBNext Multiplex Oligos for Il-lumina (Index Primers Set 2) (PN: E7500S/L; New England BioLabs, Ipswich, MA). Indexed libraries were quantitated with Qubit dsDNA HS Assay Kit (PN: Q32854; Invitrogen, Carlsbad, CA). Library quality assessment with Agilent DNA 7500 Kit (PN: 5067-1506; Agilent Technologies, Santa Clara, CA). The individual libraries were normalized, and equal molar amounts were multiplexed into a single pool. Molar concentration of the multiplex pool was determined with KAPA Library Quantification Kits for Illumina (PN: KK4824; KAPA Biosystems, Boston, MA). Materials used: NEBNext Ultra II RNA Library Prep with Sample Purification Beads (PN: E7775S/L; New England BioLabs, Ipswich, MA), NEBNext Poly(A) mRNA Magnetic Isolation Module (PN: E7490S/L; New England BioLabs, Ipswich, MA), NEBNext Multiplex Oligos for Illumina (Index Primers Set 1) (PN: E7335S/L; New England BioLabs, Ipswich, MA), NEBNext Multiplex Oligos for Illumina (Index Primers Set 2) (PN: E7500S/L; New England BioLabs, Ipswich, MA), Agilent RNA 6000 Nano Kit (PN: 5067-1511; Agilent Technologies, Santa Clara, CA). Agilent DNA 7500 Kit (PN: 5067-1506; Agilent Technologies, Santa Clara, CA), Qubit RNA HS As-say Kit (PN: Q32855; Invitrogen, Carlsbad, CA), Qubit dsDNA HS Assay Kit (PN: Q32854; Invitrogen, Carlsbad, CA), and KAPA Library Quantification Kits for Illumina (PN: KK4824; KAPA Biosystems, Boston, MA).

Sequencing. Libraries were sequenced on an Illumina NextSeq 500, paired-end, 75 total cycles to obtain greater than 20 million reads per sample. Reads were aligned to the human genome (hg19) using STAR (PMC3530905). Gene level expression was calculated (Fragments per Kilobase per Million-FPKM) using Cufflinks (PMC3146043) and differentially expressed genes identified using DE-seq (PMC3218662). For all algorithms, default parameters were utilized unless otherwise noted.

2 Supplementary Figures

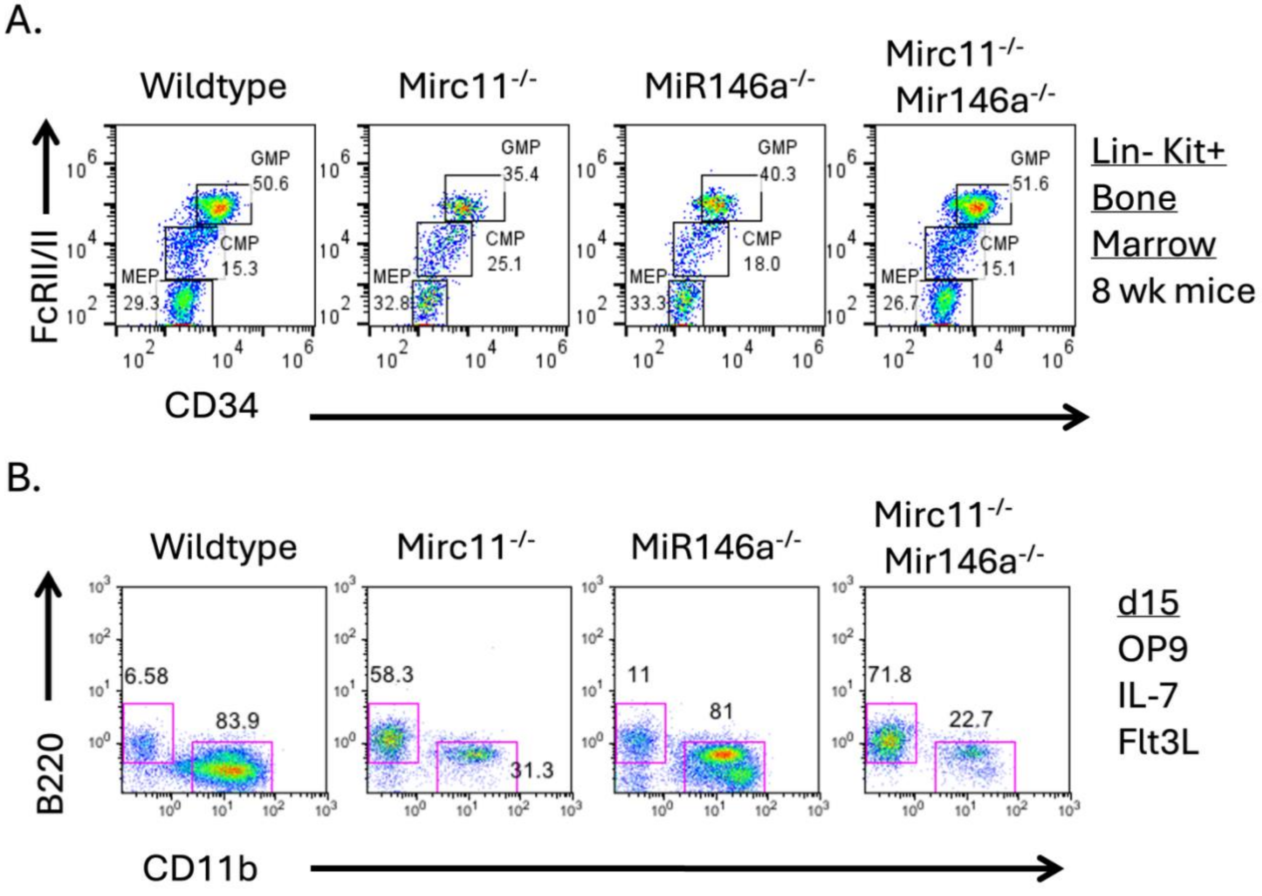

Supplementary Figure 1. Representative flow cytometric analysis of bone marrow cells isolated from 8-week-old mice. A) Nucleated bone marrow cells isolated from indicated mouse genotypes. Lineage negative bone marrow cells stained to identify myeloid progenitors. Percentage of lineage negative bone marrow is shown for each population. CMP: common myeloid progenitor, MEP: megakaryocyte-erythroid progenitor, and GMP: granulocyte-monocyte progenitor. Flow cytometric analysis performed with A) Cytek Northern Lights spectral cytometer (Fremont, CA, USA). B) Flow cytometric analysis of 15d OP9 co-cultures with lineage negative bone marrow cells isolated from indicated genotypes. Cells cultured in the presence of recombinant murine cytokines IL-7 and Flt3L. Antibodies to CD11b and B220 used to delineate myeloid (granulocytes and monocytes) and B cell populations respectively. Flow cytometric analysis was performed on with Beckman Coulter FC500 Flow Cytometer (Brea, CA, USA).

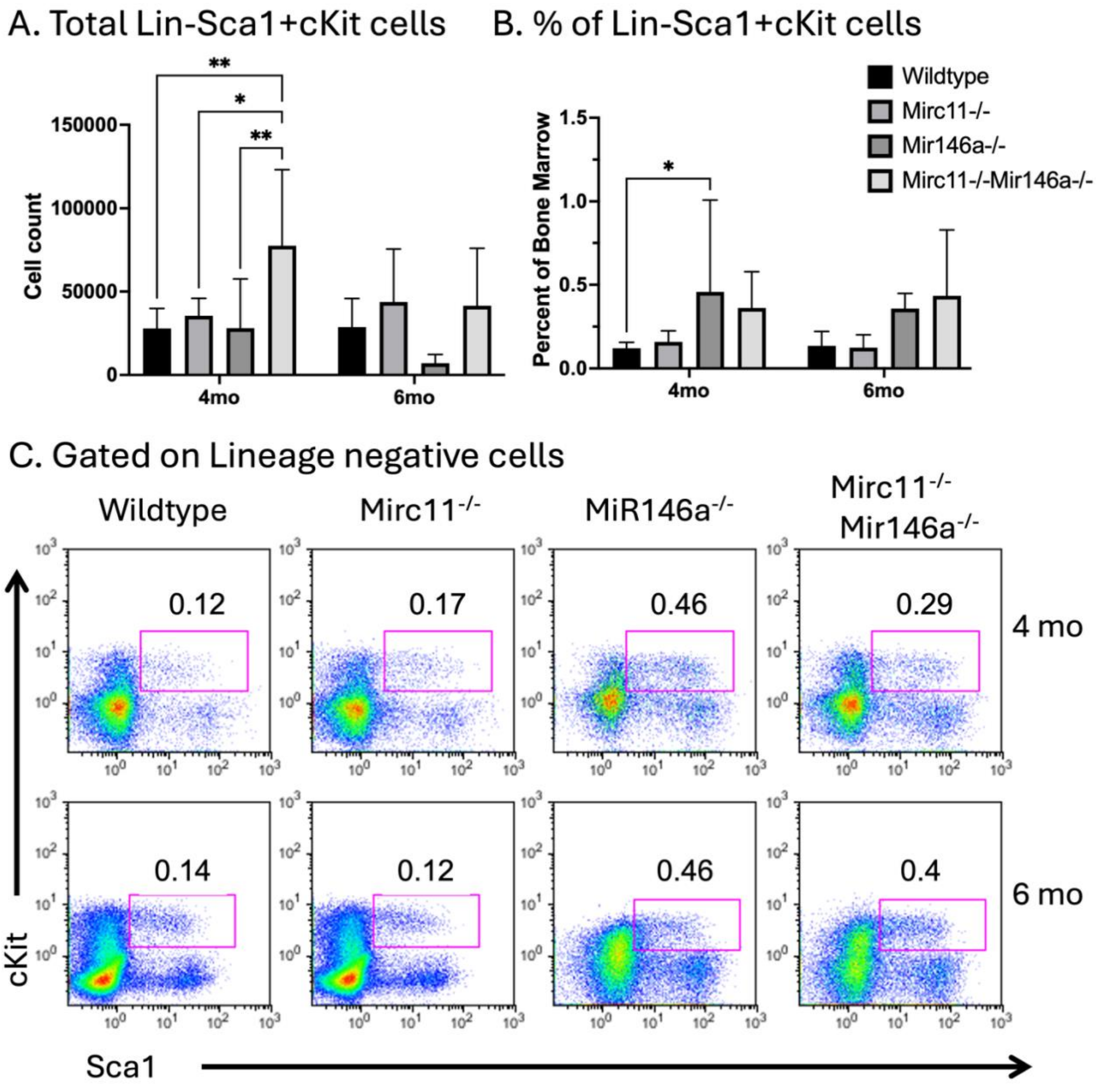

Supplementary Figure 2. Flow cytometric analysis of bone marrow LSK cells. Nucleated bone marrow isolated from mice aged 4, and 6 months (mo). A) Average total number of Lin-Sca-1+c-Kit+ HSPCs isolated from each genotype. B) Average percentage of LSKs detected in total nucleated bone marrow. Error bars denote standard deviation. *($p<0.05$), ** ($p<0.01$), *** ($p<0.001$), **** ($p<0.0001$). At least 4 mice were examined for each genotype and timepoint. C) Representative FACs plots of Sca1 vs cKit expression on nucleated bone marrow cells gated on the Lineage negative population at 4 and 6 months (mo) expression of *Nos2*, and *Il10*. Error bars denote SEM. *($p<0.05$), ** ($p<0.01$), *** ($p<0.001$). BMDM derived from 3 independent mice for each genotype were used for each assay.

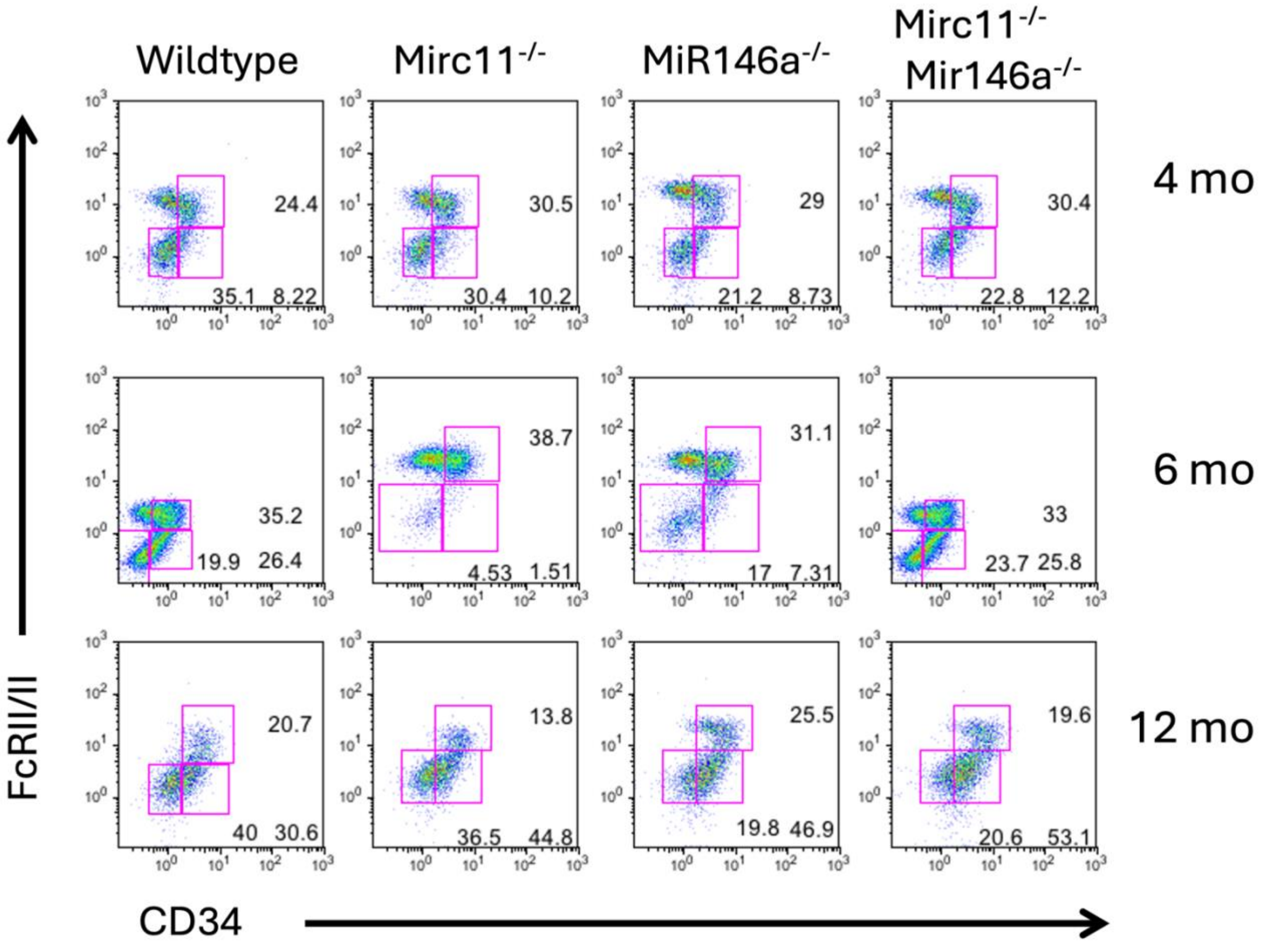

Supplementary Figure 3. Representative Flow cytometric analysis of myeloid progenitor populations in bone marrow. Nucleated bone marrow cells isolated from 4-, 6-, and 12- month (mo) old mice of the indicated genotypes. Lineage negative bone marrow cells stained to identify myeloid progenitors. Percentage of Lin-Sca1-cKit+ bone marrow is shown for each population. CMP: common myeloid progenitor, MEP: Megakaryocyte-Erythroid progenitor, and GBP: granulocyte-monocyte progenitor. Plots are generated from multiple days of analysis. Flow cytometric analysis was performed on multiple days with Beckman Coulter FC500 Flow Cytometer (Brea, CA, USA).

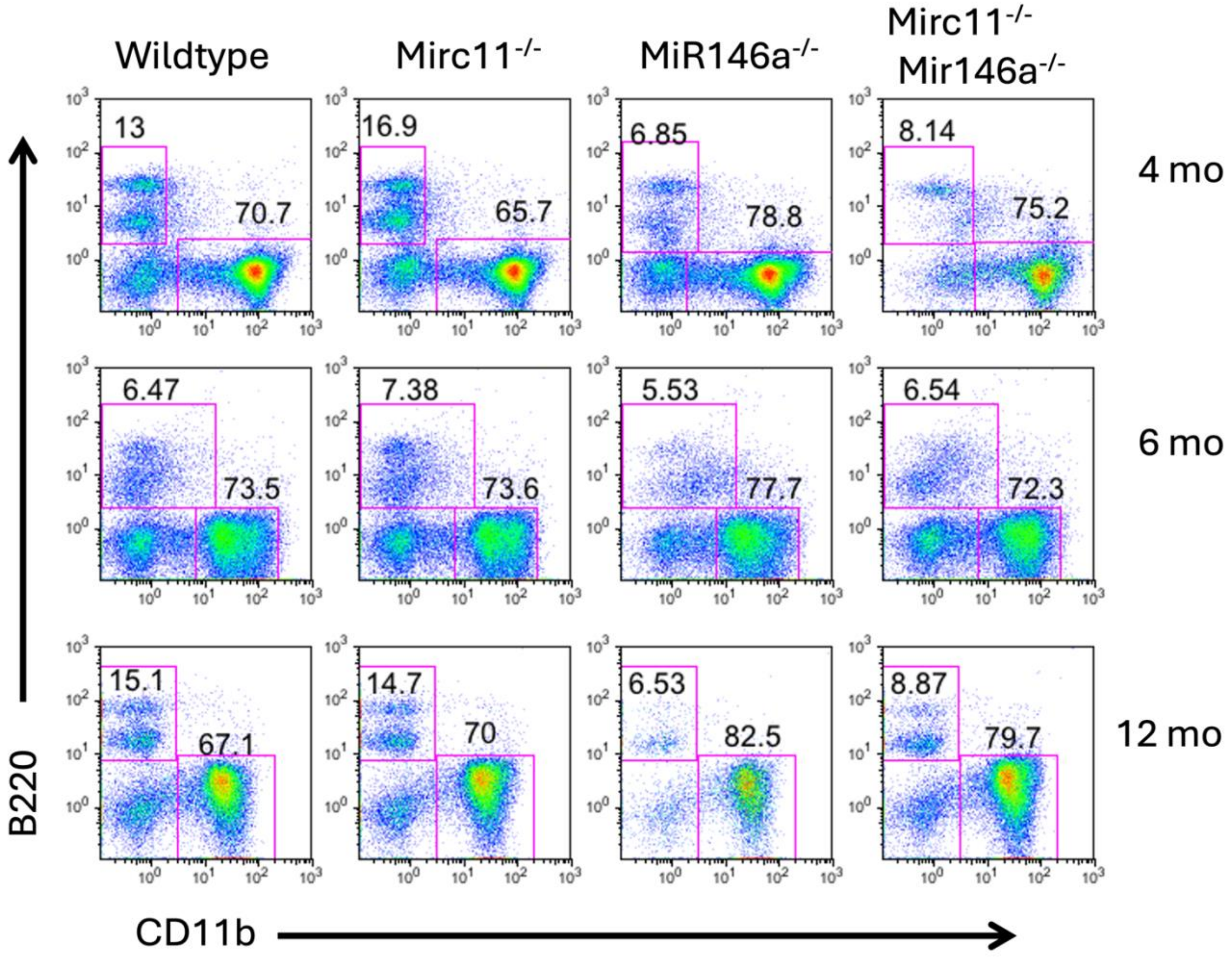

Supplementary Figure 4. Representative Flow cytometric analysis of mature myeloid and B cell populations. Nucleated bone marrow cells isolated from 4-, 6-, and 12- month (mo) old mice of the indicated genotypes. Antibodies to CD11b and B220 used to delineate myeloid (granulocytes and monocytes) and B cell populations respectively. Flow cytometric analysis was performed on multiple days with Beckman Coulter FC500 Flow Cytometer (Brea, CA, USA).

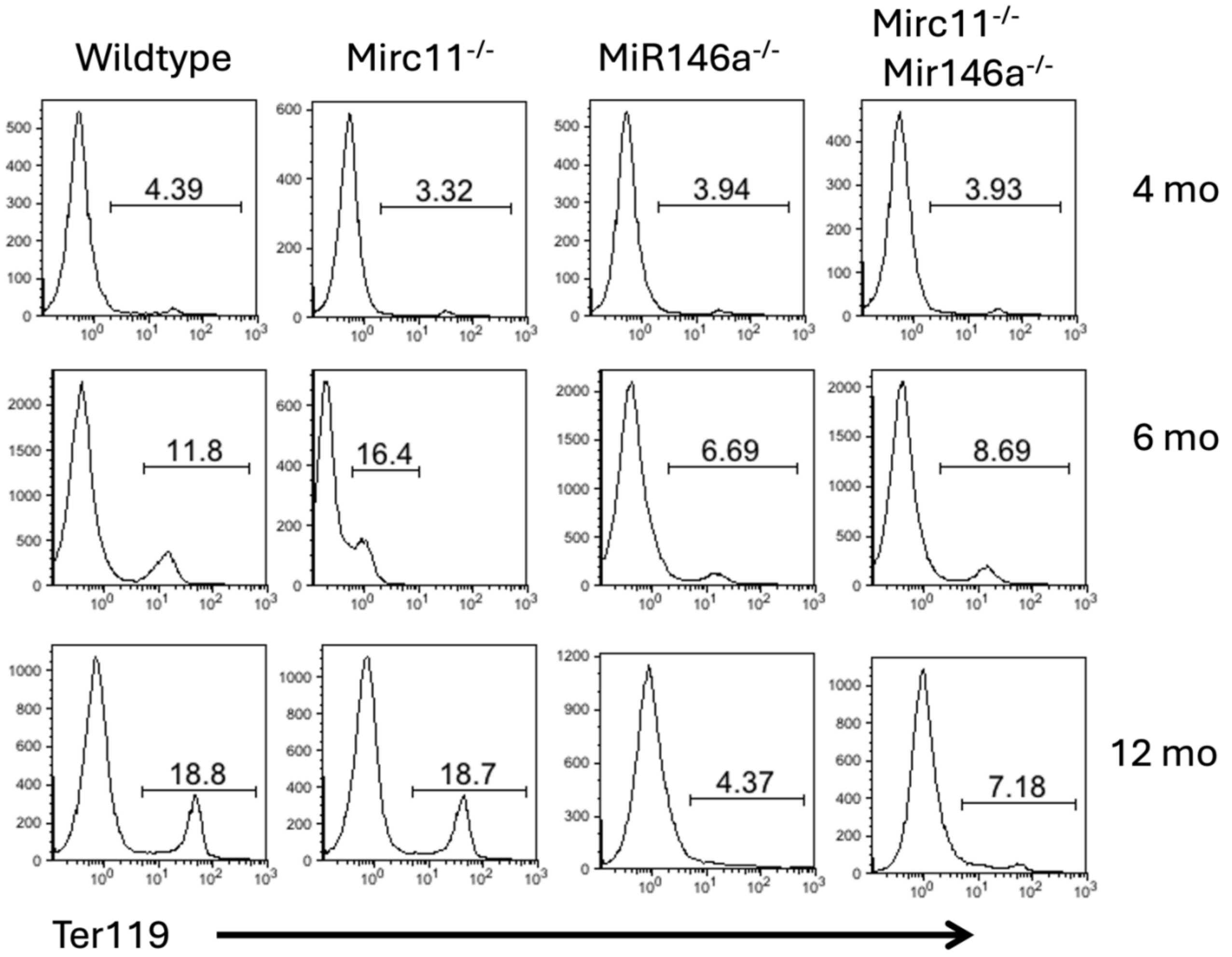

Supplementary Figure 5. Representative Flow cytometric analysis of the Ter119+ erythroid cell population. Nucleated bone marrow cells isolated from 4-, 6-, and 12- month (mo) old mice of the indicated genotypes. Antibody to Ter1119 used to identify erythroid cells. Flow cytometric analysis was performed on multiple days with Beckman Coulter FC500 Flow Cytometer (Brea, CA, USA).

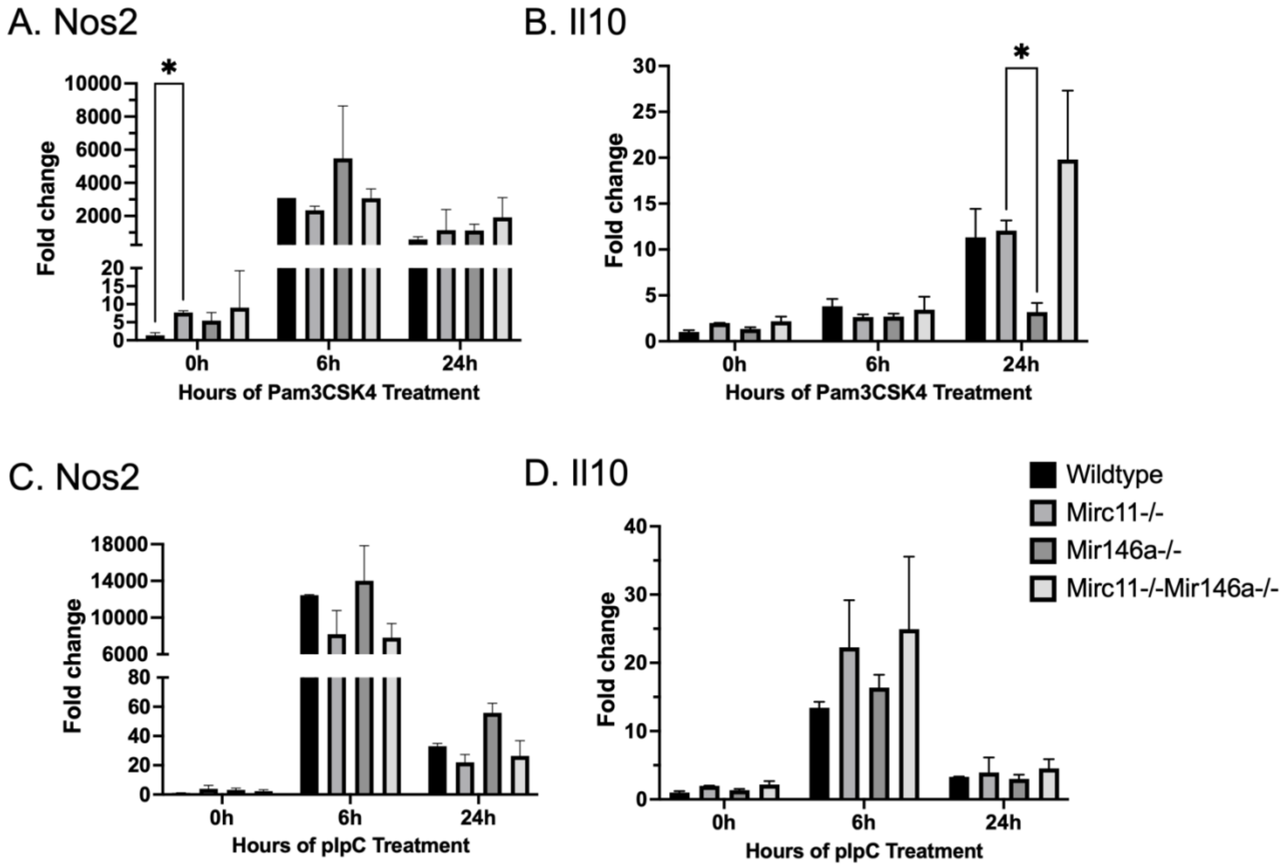

Supplementary Figure 6. Inflammatory gene expression in bone marrow derived macrophages treated with Toll like receptor (TLR) ligands. BMDMs generated from indicated genotypes were treated with A, B) 100 ng/ml Pam_3CSK_4 or C, D) 50 ng/ml pIpC for 0, 6, and 24h and assayed for expression of *Nos2*, and *Il10*. Error bars denote standard deviation. *($p<0.05$), ** ($p<0.01$), *** ($p<0.001$). BMDM derived from 3 independent mice for each genotype were used for each assay.

Supplementary Table S1: Differential expression of TNF, IL-6 and Il-1β regulated genes between wildtype and *Mirc11*$^{-/-}$ primary LSK cells determined by RNA-seq analysis.

TNF Downstream Genes			IL-6 Downstream Genes			Il-1β Downstream Genes		
**Gene**	**logFC**	**P.Value**	**Gene**	**logFC**	**P.Value**	**Gene**	**logFC**	**P.Value**
Atf3	-2.47	0.08	Atf3	-2.47	0.08	Atf3	-2.47	0.08
Mmp9	-1.56	0.00	Mmp9	-1.56	0.00	Fosb	-1.20	0.02
Klf6	-1.29	0.02	Mmp8	-1.18	0.01	Nfkbiz	-1.14	0.05
Fosb	-1.20	0.02	Fos	-1.13	0.01	Fos	-1.13	0.01
Mmp8	-1.18	0.01	Egr1	-1.10	0.05	Egr1	-1.10	0.05
Nfkbiz	-1.14	0.05	Plk2	-1.07	0.03	Ccl4	-1.07	0.03
Fos	-1.13	0.01	Ccl4	-1.07	0.03	Jun	-1.06	0.02
Plk3	-1.13	0.06	Jun	-1.06	0.02	Dusp1	-1.03	0.03
Egr1	-1.10	0.05	Dusp1	-1.03	0.03	Zfp36	-1.03	0.02
Plk2	-1.07	0.03	Jund	-0.90	0.04	Jund	-0.90	0.04
Dusp5	-1.07	0.06	Tgm2	-0.88	0.01	Junb	-0.82	0.04
Ccl4	-1.07	0.03	Ccr1	-0.85	0.01	Ier2	-0.82	0.01
Jun	-1.06	0.02	Tlr8	-0.83	0.02	Il15	-0.79	0.02
Dusp1	-1.03	0.03	Junb	-0.82	0.04	Ptgs2	-0.78	0.06
Zfp36	-1.03	0.02	Il15	-0.79	0.02	Socs1	-0.74	0.02
Cd69	-1.01	0.03	Ptgs2	-0.78	0.06	Tnfaip3	-0.73	0.06
Dusp2	-1.00	0.02	Ppp1r15a	-0.77	0.05	Pde4b	-0.72	0.05
Hspa1a	-0.96	0.02	Lpl	-0.75	0.07	Socs3	-0.72	0.03
Selp	-0.94	0.01	Socs1	-0.74	0.02	Tnf	-0.71	0.06
Jund	-0.90	0.04	Pecam1	-0.73	0.01	Zc3h12a	-0.69	0.08
Tgm2	-0.88	0.01	Socs3	-0.72	0.03	Rhob	-0.67	0.03
Ccr1	-0.85	0.01	Tnf	-0.71	0.06	Mcl1	-0.62	0.04
Tlr8	-0.83	0.02	Cybb	-0.71	0.01	Ier3	-0.60	0.02
Junb	-0.82	0.04	Lyz	-0.70	0.03	Nfkbia	-0.55	0.18
Ier2	-0.82	0.01	Apoe	-0.70	0.01	Tnfaip2	0.52	0.25
Rgs1	-0.79	0.11	Tnfrsf12a	-0.69	0.09	Lcn2	0.82	0.14
Il15	-0.79	0.02	Cd163	-0.68	0.05			
Hspa1b	-0.78	0.05	Fcgrt	-0.64	0.08			
Klf2	-0.78	0.06	Il7r	-0.64	0.09			
Ptgs2	-0.78	0.06	Ifi16	-0.64	0.00			
Ppp1r15a	-0.77	0.05	Fpr2	-0.62	0.02			
Nr4a2	-0.76	0.02	Cd68	-0.62	0.06			
Lpl	-0.75	0.07	Mcl1	-0.62	0.04			
Socs1	-0.74	0.02	Fn1	-0.61	0.01			

Scd	-0.74	0.08	Lhcgr	-0.61	0.15			
Ca12	-0.73	0.08	Il1rl1	-0.60	0.11			
Pecam1	-0.73	0.01	Cdkn1a	-0.60	0.09			
Tnfaip3	-0.73	0.06	Smad7	-0.59	0.04			
Pde4b	-0.72	0.05	Bambi	-0.57	0.10			
Socs3	-0.72	0.03	Gfap	-0.57	0.16			
Tnf	-0.71	0.06	Ceacam1	-0.56	0.15			
Cybb	-0.71	0.01	Sgk1	-0.56	0.05			
Apoe	-0.70	0.01	Prnp	-0.56	0.17			
Zc3h12a	-0.69	0.08	Pomc	-0.56	0.18			
Postn	-0.69	0.04	Nfkbia	-0.55	0.18			
Il18r1	-0.68	0.01	Cib2	-0.54	0.20			
Cd163	-0.68	0.05	Tbc1d9	-0.53	0.18			
Nqo1	-0.68	0.03	Lcat	-0.53	0.24			
Cnr2	-0.68	0.09	Acp5	-0.52	0.25			
Rhob	-0.67	0.03	Lcn2	0.82	0.14			
Rgs2	-0.67	0.01						
Aldh1a7	-0.66	0.20						
Oasl	-0.65	0.03						
Lgals3	-0.65	0.02						
Ghr	-0.65	0.12						
Fcgrt	-0.64	0.08						
Il7r	-0.64	0.09						
Sdc1	-0.64	0.09						
Ifi16	-0.64	0.00						
Klf4	-0.64	0.02						
Mx1	-0.63	0.09						
Cyp27a1	-0.62	0.14						
Fpr2	-0.62	0.02						
Mcl1	-0.62	0.04						
Slc11a2	-0.62	0.10						
Emp1	-0.61	0.11						
Agtr1	-0.61	0.23						
Fn1	-0.61	0.01						
Lhcgr	-0.61	0.15						
Sorbs1	-0.61	0.12						
Btg1	-0.60	0.15						
Cyth3	-0.60	0.12						
Ier3	-0.60	0.02						
Il1rl1	-0.60	0.11						
Cdkn1a	-0.60	0.09						
Pou2af1	-0.60	0.13						

Smad7	-0.59	0.04						
Sdc2	-0.59	0.12						
Mst1r	-0.59	0.13						
Adrb2	-0.59	0.11						
Pla2g4c	-0.59	0.10						
Trem2	-0.58	0.03						
Pim3	-0.58	0.11						
Nov	-0.58	0.19						
Ca2	-0.58	0.21						
Trpc6	-0.58	0.15						
Oas2	-0.58	0.06						
Bambi	-0.57	0.10						
Gfap	-0.57	0.16						
Ampd3	-0.57	0.14						
Mfhas1	-0.56	0.13						
Kcnj2	-0.56	0.15						
Cdk5r1	-0.56	0.14						
Sgk1	-0.56	0.05						
Prnp	-0.56	0.17						
Ifit3	-0.56	0.16						
Pomc	-0.56	0.18						
Rcn3	-0.55	0.16						
Nfkbia	-0.55	0.18						
Jag2	-0.55	0.19						
Hid1	-0.55	0.21						
Rnase4	-0.54	0.18						
Cib2	-0.54	0.20						
St3gal5	-0.54	0.17						
Il17rb	-0.54	0.17						
Lrig1	-0.53	0.24						
Lcat	-0.53	0.24						
Ccl9	-0.53	0.22						
Atf4	-0.53	0.00						
Acp5	-0.52	0.25						
Alad	-0.51	0.30						
Bhlhe40	-0.50	0.12						
Optn	0.50	0.13						
Tnfaip2	0.52	0.25						
Lcn2	0.82	0.14						
